# A full vectorial mapping of nanophotonic light fields

**DOI:** 10.1038/s41377-019-0124-3

**Published:** 2019-03-06

**Authors:** B. le Feber, J. E. Sipe, M. Wulf, L. Kuipers, N. Rotenberg

**Affiliations:** 1 Optical Materials Engineering Laboratory, ETH Zürich, 8092 Zurich, Switzerland; 2 Center for Nanophotonics, AMOLF, Science Park 104, 1098 XG Amsterdam, The Netherlands; 3 Institute for Optical Sciences, University of Toronto, 60 St. George Street, Ontario, M5S 1A7 Canada; 4 Institute of Science and Technology Austria, Am Campus 1, 3400 Klosterneuburg, Austria; 5 Kavli Institute of Nanoscience, Department of Quantum Nanoscience, Delft University of Technology, Lorentzweg 1, 2628 CJ Delft, The Netherlands; 6 Niels Bohr Institute and Center for Hybrid Quantum Networks, University of Copenhagen, Blegdamsvej 17, DK-2100 Copenhagen, Denmark

**Keywords:** Nanophotonics and plasmonics, Imaging and sensing, Photonic crystals, Scanning probe microscopy

## Abstract

Light is a union of electric and magnetic fields, and nowhere is the complex relationship between these fields more evident than in the near fields of nanophotonic structures. There, complicated electric and magnetic fields varying over subwavelength scales are generally present, which results in photonic phenomena such as extraordinary optical momentum, superchiral fields, and a complex spatial evolution of optical singularities. An understanding of such phenomena requires nanoscale measurements of the complete optical field vector. Although the sensitivity of near-field scanning optical microscopy to the complete electromagnetic field was recently demonstrated, a separation of different components required a priori knowledge of the sample. Here, we introduce a robust algorithm that can disentangle all six electric and magnetic field components from a single near-field measurement without any numerical modeling of the structure. As examples, we unravel the fields of two prototypical nanophotonic structures: a photonic crystal waveguide and a plasmonic nanowire. These results pave the way for new studies of complex photonic phenomena at the nanoscale and for the design of structures that optimize their optical behavior.

The advent of metamaterials and structures with a large response to the optical magnetic field ushered in a new age of near-field microscopy, where the ability to measure only electric near fields is no longer sufficient. Many nanoscopic structures, such as split ring resonators^1,2^, dielectric Mie scatterers^3–6^, and even simple plasmonic holes^7,8^, have an optical response that depends on the full electromagnetic field. Likewise, measurements of many nanoscale photonic phenomena, such as superchiral fields^9,10^ or extraordinary spin and orbital angular momentum^11–13^, require access to both the electric **E** and magnetic **H** fields.

Motivated by this demand, there have been a number of efforts to extend the capability of near-field scanning optical microscopes (NSOMs) beyond the traditional measurements of **E**^14^. Proof-of-concept measurements of **H** at the nanoscale have relied on specially designed near-field probes^15,16^; however, these are difficult to fabricate and tend to measure only one component of **H**. Recent strategies have therefore focused on measurements with traditional aperture probes^17,18^, which demonstrate that even circular apertures are simultaneously sensitive to the four in-plane components $E_{x,y}$ and $H_{x,y}$ ^19^.

However, a crucial challenge remains. Although a polarization-resolved NSOM measurement (see Supplementary Note 1) contains information from the four in-plane components, it is encoded into only two complex signals *L*_*x*_ and *L*_*x*_, as shown in Fig. 1. To date, unraveling these measurements to extract the individual components of the electric and magnetic fields has not been possible without the use of additional information coming from detailed simulations of the structure being measured^20^, on far-field optical beams^21^ or a symmetry plane where one component is identically zero^22^. At best, numerical simulations can be used to determine the spatial evolution of $\left| {\mathbf{E}} \right|^2$ and $\left| {\mathbf{H}} \right|^2$ near nanophotonic structures but not separate electromagnetic components or their phases^23^. Here, we show how to simultaneously extract $E_x$, $E_y$, $H_x$, and $H_y$ from a single two-channel NSOM measurement with no a priori knowledge of the nanophotonic structures being measured. By inserting these fields into Maxwell’s equations, we can obtain the two out-of-plane components $E_z$ and $H_z$ and thus achieve a full vectorial measurement of the electromagnetic near-field. The separation algorithm is robust to noise and realistic measurement conditions, as we show from exemplary NSOM experiments on both photonic crystal waveguides (PhCWs) and plasmonic nanowires.

**Fig. 1:**
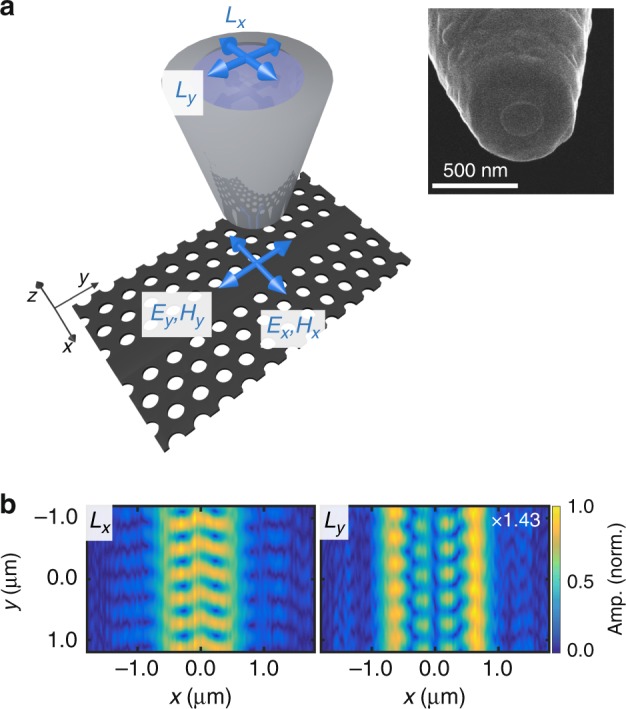
Polarization-resolved near-field measurements. **a** Sketch of the essentials of the polarization-sensitive NSOM used in this work. The blue arrows near the sample indicate the electric and magnetic fields along *x* and *y*. The probe converts these fields to radiation polarized along *x* and *y*, as indicated by the top blue arrows. The inset shows an SEM of the aperture probe used for the photonic crystal waveguide measurements. **b** Two-dimensional maps of the amplitude of $L_x$ (left panel) and $L_y$ (right) measured by raster-scanning the tip 280 nm above the photonic crystal waveguide

At the heart of near-field microscopy lies the process by which the near-field probe images the light fields above a structure. For example, in the field distributions in Fig. 1b, which were measured 280 nm above a PhCW (Supplementary Notes 1 and 2), a representative height where the electric and magnetic field distributions contain subwavelength features and are expected to differ^19,24^. These images are produced as the aperture probe, which acts as an effective spatial filter, merges all four in-plane components of the sample’s near-field. When this light field is highly structured with feature sizes smaller than the probe aperture, this process becomes increasingly complex, and it is less obvious exactly how efficiently and with what phase $E_x$, $E_y$, $H_x$, and $H_y$ contribute to the measured signals $L_y$ and $L_x$. In other words, calculating the transfer function of a near-field probe, which propagates the fields from the sample to a detector, has not been possible.

However, it is possible to calculate the fields that are radiated through the probe by a point dipole at position **r**_0_ of a hypothetical detector (Fig. 2a) with current density ${\mathbf{j}}_{{\rm{det}}}{\mathrm{\delta }}({\mathbf{r}} - {\mathbf{r}}_0)$. These fields, which we label ${\mathbf{E}}_i^r$ and ${\mathbf{H}}_i^r$, where  *i *= *x,y* indicates the orientation of **j**_det_ (Fig. 2b, middle column, for the dipole in the *x* direction), have been extensively measured and resemble those below a hole in a metal film^14,25^; hereafter, we take our tip to be ideally symmetric to ensure equal sensitivity to the *x* and *y* components of the electromagnetic field. Via the optical reciprocity theorem (ORT), we can use these probe fields to relate the sample fields ${\mathbf{E}}^e$ and ${\mathbf{H}}^e$ (Fig. 2b, left column) to dipoles at our detectors and, consequently, our measured signals (Fig. 2b, right column)^19,26,27^. In other words, in this approach, ${\mathbf{E}}_i^r$ and ${\mathbf{H}}_i^r$ can be considered the spatial filters that exactly define how efficiently and with which phase different sample field components are detected. Each independent dipole orientation *x* or *y* is associated with all four in-plane components of the probe field, which explains why each detection channel typically contains information of all in-plane components of the sample fields. Using a specific sensing configuration^28^ or material composition^16^, it is possible to design probes that primarily detect ${\mathbf{E}}^e$ or ${\mathbf{H}}^e$ of specific near fields. However, such probes preclude complete electromagnetic measurements, so we consider aperture probes that are similarly sensitive to ${\mathbf{E}}^e$ and ${\mathbf{H}}^e$ in this work.

**Fig. 2:**
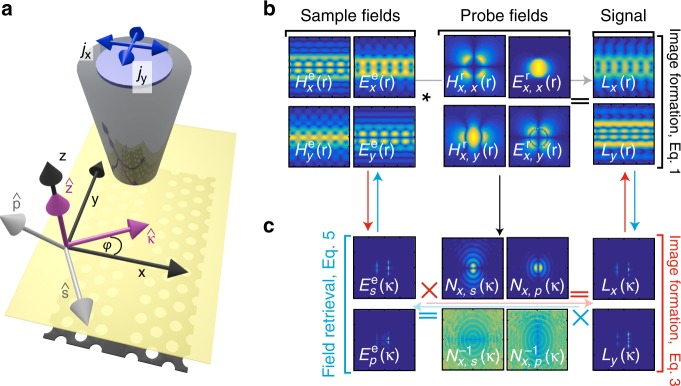
Image formation and field retrieval. **a** Schematic of the coordinate bases and experimental setup. All fields are evaluated on a surface (transparent yellow) that completely separates the probe from the sample. The purple arrows indicate the in-plane (${\hat{\mathbf \kappa }}$) and out-of-plane (${\hat{\mathbf z}}$) unit vectors of a plane wave on this surface, whereas the gray arrows show the corresponding unit vectors ${\hat{\mathbf s}}$ and ${\hat{\mathbf p}}$ for an upward traveling wave. **b** Real space image formation process according to Eq. 1. In real space, the measured image $L_{x,y}$ can be understood as the convolution (indicated by the asterisk sign) of the sample fields ${\mathbf{E}}^e$ and ${\mathbf{H}}^e$ and the probe fields ${\mathbf{E}}_i^r$ and ${\mathbf{H}}_i^r$, shown here for the *x*-oriented dipole (*i* = *x*). **c** Top row: In Fourier space, the image formation process that corresponds to (**b**) is described by the multiplication of the sample fields and probe response function ${\rm{N}}$, which was here calculated for an ideally symmetric probe. Bottom row: The reverse process, which results in the separated fields, therefore simply involves the multiplication of the measured signals with the inverse probe response function ${\rm{N}}^{ - 1}$. Note that we only show only the *x*-oriented dipole (*i* = *x*) components of ${\rm{N}}$ and ${\rm{N}}^{ - 1}$. All maps in (**b**, **c**) show the calculated fields that are normalized to their maximum amplitude

The image formation via the ORT can be expressed as (see Supplementary Note 4 for derivation)

1 $$L_i\left( {{\mathbf{R}}_{{\rm{tip}}}} \right) =	 {\int_{S}} dS\left( {\mathbf{E}}^e\left( {\mathbf{R}} \right) \times {\mathbf{H}}_i^r\left( {{\mathbf{R}} - {\mathbf{R}}_{{\rm{tip}}}} \right)\right. \\ 	- \left.{\mathbf{E}}_i^r\left( {{\mathbf{R}}} - {{\mathbf{R}}_{{\rm{tip}}}} \right) \times {\mathbf{H}}^e\left( {\mathbf{R}} \right) \right) \cdot {\hat{\mathbf z}}$$

where *S* is a surface between the probe and the sample, which is 10 nm below the probe in this case; ${\mathbf{R}}_{{\rm{tip}}} = \left( {x_{{\rm{tip}}},y_{{\rm{tip}}}} \right)$ is the position of the tip above this plane; ${\mathbf{R}} = \left( {x,y} \right)$ are the coordinates of the fields on *S*; and the integral is taken over all **R**. Subscript *i* refers to the *x* or *y* orientation of the reciprocal dipole and not to a component of the fields. The dot product with ${\hat{\mathbf z}}$ shows that the measured image only depends on the in-plane field components. This process of image formation is shown in Fig. 2b, where we use the calculated probe and sample fields to predict the measured signals (see ref. ^19^ for details on the calculations). In fact, we observe an excellent agreement between our predictions (right column, Fig. 2b) and the measurements (Fig. 1b) at 280 nm above the PhCW, which validates this approach and the symmetry of our probes.

When we want to retrieve the sample fields, instead of studying the image formation, we face two challenges: first, we require two additional equations to match the number of unknowns; second, we must be able to invert Eq. 1 (Supplementary Note 3). To address the first challenge, we recognize that the electromagnetic field at and near the sample plane can be decomposed into a superposition of different plane waves, each of which is represented by a total wavevector ${\mathbf{k}} = k_z{\hat{\mathbf z}} + \varkappa {\hat{\mathbf \kappa }}, {\rm{where}}{\,} \varkappa=|\kappa|=|(k_x, k_y)|$^28^. Here, $k_z$ is the out-of-plane component of the wavevector, and ${\mathbf{\varkappa }}$ is the in-plane component, as shown in Fig. 2a. We can write each plane wave in the Cartesian basis (*E*_*x*_, *E*_*y*_, *E*_*z*_*)* or in terms of its *s*- and *p*-field components ($E_{s + }$, $E_{s - }$, $E_{p + }$, $E_{p - }$), which enables us to identify the upward (real (*k*_*z*_) > 0, subscript +) or downward (real (*k*_*z*_) < 0, subscript −) propagating waves. In principle, the full field between the sample and the probe is a combination of both upward and downward propagating fields, where the latter arise due to the interaction of the probe tip with the sample. However, this interaction is negligible if the probe and sample do not have a joint resonance^29^, as is indeed the case for our normal aperture probe, which has a broad spectral response^7,28^. Therefore, we can take the field above the sample surface to be purely upward propagating (i.e., there is no backscattering, so $E_{p - } = 0$), which implies that we must only consider two components of the electric field and four components of the total field: *E*_*s*_, *E*_*p*_, *H*_*s*_, and *H*_*p*_, where all *s* and *p* components are upward propagating (i.e., *p* + ). Finally, Maxwell’s equations straightforwardly relate the electric and magnetic field components of these transverse plane waves (see Supplementary Note 5 for the derivation and conversion between the different bases)

2 $$\begin{array}{l}{\mathbf{E}}^e({\mathbf{\kappa }}) = E_s^e({\mathbf{\kappa }}){\hat{\mathbf s}} + E_p^e({\mathbf{\kappa }}){\hat{\mathbf p}},\\ {\mathbf{H}}^e({\mathbf{\kappa }}) = \frac{1}{{Z_0}}\left[ {E_s^e({\mathbf{\kappa }}){\hat{\mathbf s}} - E_p^e({\mathbf{\kappa }}){\hat{\mathbf p}}} \right]\end{array}$$

where *Z*_0_ is the impedance of free space. Considering Eq. 1, we have reduced our problem to two unknowns ($E_s^e$ and $E_p^e$) and two equations, one each for *L*_*x*_ and *L*_*y*_. In terms of the Fourier components, we can rewrite Eq. 1 as

3 $$\frac{1}{{Z_0}}\left[ {\begin{array}{*{20}{c}} {L_x\left( {\mathbf{\kappa }} \right)} \\ {L_y\left( {\mathbf{\kappa }} \right)} \end{array}} \right] = \left[ {\begin{array}{*{20}{c}} {N_{x,s}\left( {\mathbf{\kappa }} \right)} & {N_{x,p}\left( {\mathbf{\kappa }} \right)} \\ {N_{y,s}\left( {\mathbf{\kappa }} \right)} & {N_{y,p}\left( {\mathbf{\kappa }} \right)} \end{array}} \right]\left[ {\begin{array}{*{20}{c}} {E_s^e\left( {\mathbf{\kappa }} \right)} \\ {E_p^e\left( {\mathbf{\kappa }} \right)} \end{array}} \right]$$

where tensor ${\mathrm{N}}$ is essentially the transfer matrix that maps the sample electric fields expressed in their polarization components to the detection channels associated with the $x$- and $y$-directions. Different components of ${\rm{N}}$ are related to the Cartesian components of ${\mathbf{E}}_i^r$ and ${\mathbf{H}}_i^r$ as follows

4 $$	{{\hskip-1.4pc}\left[ {\begin{array}{*{20}{c}} {N_{i,s}\left( {\mathbf{\kappa }} \right)} \\ {N_{i,p}\left( {\mathbf{\kappa }} \right)} \end{array}} \right]} \\ 	{{\hskip-1.4pc} = \left[ {\begin{array}{*{20}{c}} { - \frac{{k_z}}{{k_0}}\sin \varphi } & {\!\!} {\frac{{k_z}}{{k_0}}\cos \varphi } &{\!\!} {Z_0\cos \varphi } &{\!\!}{\!\!} {Z_0\sin \varphi } \\ {\cos \varphi } & {\!\!} {\sin \varphi } &{\!\!} {Z_0\frac{{k_z}}{{k_0}}\sin \varphi } & {\!\!}{ - Z_0\frac{{k_z}}{{k_0}}\cos \varphi } \end{array}} \right] \left[ {\begin{array}{*{20}{c}} {E_{i,x}^r\left( { - {\mathbf{\kappa }}} \right)} \\ {E_{i,y}^r\left( { - {\mathbf{\kappa }}} \right)} \\ {H_{i,x}^r\left( { - {\mathbf{\kappa }}} \right)} \\ {H_{i,y}^r\left( { - {\mathbf{\kappa }}} \right)} \end{array}} \right]}$$

where $\varphi$ is the angle between ${\mathbf{\kappa }}$ and the *x*-axis (Fig. 2a). We show the image formation process in terms of these plane wave components in the top row of Fig. 2c, which corresponds to the real space plots in Fig. 2b, where $N_{x,s}\left( {\mathbf{\kappa }} \right)$ and $N_{x,p}\left( {\mathbf{\kappa }} \right)$ are plotted in the middle column. These N maps clearly show which wavevector components contribute the most to the detected image.

Then, unraveling the near-field measurements is simply a matter of inverting N to obtain

5 $$\left[ {\begin{array}{*{20}{c}} {E_s^e\left( {\mathbf{\kappa }} \right)} \\ {E_p^e\left( {\mathbf{\kappa }} \right)} \end{array}} \right] = \frac{1}{{Z_0}}\left[ {\begin{array}{*{20}{c}} {N_{x,s}^{}\left( {\mathbf{\kappa }} \right)} & {N_{x,p}^{}\left( {\mathbf{\kappa }} \right)} \\ {N_{y,s}^{}\left( {\mathbf{\kappa }} \right)} & {N_{y,p}^{}\left( {\mathbf{\kappa }} \right)} \end{array}} \right]^{ - 1}\left[ {\begin{array}{*{20}{c}} {L_x\left( {\mathbf{\kappa }} \right)} \\ {L_y\left( {\mathbf{\kappa }} \right)} \end{array}} \right]$$

which has a unique solution if ${\rm{det}}\left( {\rm{N}} \right) \ne 0$ for all ${\mathbf{\kappa }}$, as is indeed the case for our probes. Therefore, we can deconvolve a near-field measurement simply by following the steps illustrated in the bottom row of Fig. 2c. First, the measurements are Fourier transformed in the *xy*-plane to generate $L_{x,y}\left( {\mathbf{\kappa }} \right)$, which are multiplied by ${\rm{N}}^{ - 1}\left( {\mathbf{\kappa }} \right)$ to obtain $E_{s,p}^e\left( {\mathbf{\kappa }} \right)$ according to Eq. 5. Then, these fields are transformed back into the Cartesian basis (Supplementary Note 5) and inverse-Fourier-transformed into the real space to arrive at the deconvolved sample fields $E_{x,y}^e\left( {\mathbf{R}} \right)$ and $H_{x,y}^e\left( {\mathbf{R}} \right)$. Finally, following the example of Olmon et al.^22^, we use Maxwell’s equations to extract the 2D maps of the out-of-plane electric and magnetic field components, $E_z^e({\mathbf{R}})$ and $H_z^e({\mathbf{R}})$, according to $E_z = iZ_0k_0\left( {\frac{{\partial H_x}}{{\partial x}} - \frac{{\partial H_x}}{{\partial y}}} \right)$ and $H_z = - \frac{{ik_0}}{{Z_0}}\left( {\frac{{\partial E_y}}{{\partial x}} - \frac{{\partial E_x}}{{\partial y}}} \right)$. Because the same probe can be used for multiple measurements and ${\mathrm{N}}\left( {\mathbf{\kappa }} \right)$ is similar for probes with different aperture sizes (Supplementary Figure S13), ${\rm{N}}^{ - 1}\left( {\mathbf{\kappa }} \right)$ must only be calculated once and can be used in many experiments.

The inversion of N (in Eq. 5) makes our deconvolution process sensitive to large-wavevector signals, although the image formation process is not (bottom and top rows of Fig. 2c, respectively). Since the experimental fields (left column, Fig. 2c) do not contain a signal at these large wavevectors, the measurement noise typically dominates there. In principle, this sensitivity to large wavevectors limits our retrieval algorithm, but it does not greatly affect its performance in practice. As we discuss below (see Fig. 4), we can simply limit the largest wavevector that we consider to the wavevector at which we still expect to find signal from the sample.

Here, we apply our algorithm to the PhCW fields shown in Fig. 1b and limit ourselves to the smallest allowable wavevector range $\varkappa \le 3k_0$, where *k*_0_ is the free-space wavenumber of the light to test our retrieval procedure in the lowest resolution limit. As we discuss below and in more detail in Supplementary Note 7, the maximum allowable wavevector can by increased to $\varkappa _{{\mathrm{max}}} = 9k_0$. The amplitudes of the separated field components are shown in Fig. 3 along with the theoretically calculated mode profiles. Line cuts, taken at the positions of the dashed lines are also shown, which demonstrate the excellent agreement when comparing the experimental (blue) and theoretical (gray) curves for all six electromagnetic field components. In fact, the only component for which we observe significant deviation between the predicted and measured field amplitude is *E*_*z*_. We attribute this difference to the small amplitude of this component, which makes it more susceptible to errors that arise from imperfect experimental conditions, which can cause, e.g., polarization mixing. In principle, even these small errors can be improved by calculating the transfer function for the exact probe used, including minor fabrication imperfections, and not the idealized, symmetric probe here. We also observe strikingly good agreement between the calculated and retrieved phase profiles (Supplementary Figure S14). In other words, we can successfully recover the general shape of each field component and even resolve the fine features in the amplitude and phase of these in-plane fields all from a single measurement.

**Fig. 3:**
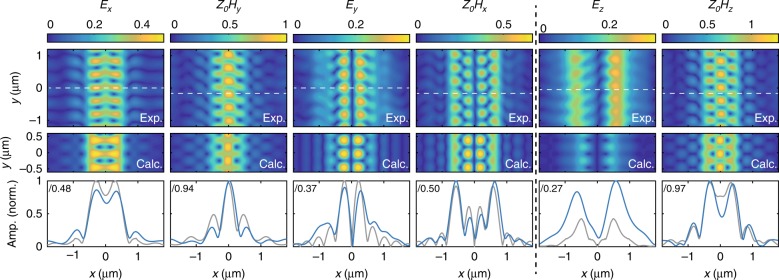
Retrieved PhCW electric and magnetic fields. The panels show two-dimensional amplitude maps of the retrieved (top) and calculated (middle) electric and magnetic fields at 280 nm above a PhCW. The field components shown in each column are indicated above that column, where the black dashed line separates the in- and out-of-plane fields. The retrieved and calculated amplitudes are normalized to the maximum amplitude of the retrieved $H_{y}$. In the bottom row of the panels, we show line cuts taken across the maxima of each field, as indicated by the white dashed lines in the field maps. Blue and gray lines correspond to line cuts through the retrieved and calculated fields, respectively. To show all fields on the same axis, we scaled the amplitude with the factors shown in the top left of each panel

Our approach is not limited to dielectric structures but can be extended to nanoplasmonics. As an example, we consider a plasmonic nanowire, whose electric and magnetic near-field distributions are known to have different and nontrivial spatial dependencies^30^. Using our protocol, we resolve the different field components above the nanowire (see Supplementary Note 6 for details and images of the separated fields). We again observe good agreement between theoretical and measured fields, and similar to the dielectric samples, clear differences in the retrieved electric and magnetic fields from different samples are revealed (Supplementary Figure S10).

The ability of our algorithm to retrieve optical fields from measurements of a PhCW and a plasmonic nanowire already hints at its robustness to noise. To further explore the effect of measurement noise, we artificially add white noise to a perfect “measurement” (i.e., theoretically calculated fields with a noise level < 10^−3^) in increments until we reach a signal-to-noise ratio of unity in *L*_*x,y*_. Then, we calculate the normalized error between the ideal and the retrieved optical fields (see Methods Section), which is shown in Fig. 4. More importantly, for all noise levels, we observe that the setting $\varkappa _{\max } = 2k_0$ results in a poor field retrieval because this low limit effectively filters large portions of the input signal (Supplementary Figure S12 for the corresponding retrieved field maps and Supplementary Section S7 for additional discussion). However, up to $\varkappa _{{\rm{max}}} = 5k_0$, we find near-perfect deconvolution even in cases where the noise is as large as the signal.

**Fig. 4:**
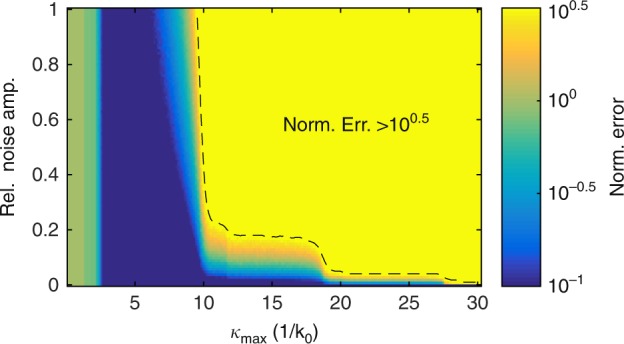
Robustness of the field retrieval algorithm. Mismatch between the retrieved fields and the predicted fields (see Methods) as a function of the noise amplitude and wavevector cutoff (see text for explanation). Because small signals with a high spatial frequency can result in very large signals, which are well beyond the total intensity of the calculated fields, we saturate Fig. 4 at normalized errors larger than 10^0.5^ to avoid obscuring more important results at low mismatch values. Likewise, the minimum error in our calculations is at 10^−5^; because all values below 10^−1^ practically appear identical to the input fields, we saturated this plot below 10^−1^

Finally, we note that while decreasing the probe aperture size results in a decrease in signal and a corresponding increase in resolution, it has little effect on our algorithm (Supplementary Figure S13); although higher wavevectors appear in ${\rm{N}}\left( {\mathbf{\kappa }} \right)$ for small probe diameters, ${\rm{N}}\left( {\mathbf{\kappa }} \right)$ remains nearly identical at low ${\mathbf{\varkappa }}$. Since the algorithm is robust even when the noise level is comparable to the signal (c.f. Figure 4), even measurements with such low-throughput probes can be deconvolved into their constituent components.

The capability to map both the electric and magnetic near-field components is important for the study and development of nanophotonic structures, particularly if the strategy is simple and robust. Our approach can be used to measure the full electric and magnetic fields near dielectric and plasmonic structures, which are increasingly necessary in a research landscape of nanoscopic structures with different electric and magnetic responses. Moreover, because the deconvolution of a full field takes only seconds when ${\rm{N}}^{ - 1}\left( {\mathbf{\kappa }} \right)$ is known (Eqs. 4 and 5), our algorithm can be applied in real time. As a demonstration, we have presented the full, complex electromagnetic near-field of two nanophotonic waveguides, but we note that our approach can also be applied to other systems such as nanoantennas and cavities. For the latter case, special care must be taken with high-quality factor resonators $Q \, > \, 1000$, where the interactions between the near-field probe and the photonic mode cannot be neglected and in fact can provide an independent measure of the magnetic field^17,31^. Measurements of nanoscale **E** and **H** have the potential to drive progress in fields such as chiral quantum optics^32,33^, plasmonics^34^, and metasurfaces^35^, where the light-matter interactions and device performance depend on the exact form of vector near fields, often in the presence of unavoidable fabrication imperfections. A further intriguing possibility is the combination of our method with measurements of the emission of a quantum emitter placed on the probe, which map out the local density of optical states^36,37^ and are therefore important to quantum optical applications.

## Methods

### Robustness to noise

To quantify the robustness to noise of our algorithm, we compare the calculated fields to the fields retrieved from a computer-generated field map, which is obtained by applying the reciprocity theorem to the calculated fields. To this calculated mapping (such as that in Fig. 2b), we add a controlled amount of white noise. The mean amplitude of that noise relative to the maximum amplitude of the signal is shown on the *y*-axis of Fig. 4. Next, we apply our algorithm to these noisy calculated mappings and compare the retrieved fields to the calculated fields to obtain the normalized error $\Delta = \mathop {\sum}\limits_{E_{x,y}H_{x,y}} {{\int} {\left| {\left| {F_{{\rm{retr}}}} \right| - \left| {F_{{\rm{in}}}} \right|} \right|^2{\rm{dr}}} } /\mathop {\sum}\limits_{E_{x,y}H_{x,y}} {{\int} {\left| {F_{{\rm{in}}}} \right|^2{\rm{dr}}} }$, where *F* indicates the electric and magnetic field components of the retrieved (retr.) and input (in) fields.

### SP coordinate transformations

The orientation of the *sp*-basis vectors is constructed from the in-plane wavevector according to

6 $${\hat{\mathbf s}} = {\hat{\mathbf \kappa }} \times {\hat{\mathbf z}}$$

7 $${\hat{\mathbf p}}_ \pm = \frac{{\varkappa {\hat{\mathbf z}} \mp k_z{\hat{\mathbf \kappa }}}}{{k_0}}$$

where $k_z = \sqrt {k_0^2 - \varkappa ^2}$. In our experiment, there are only upward-propagating fields, and we use the following equations to convert the fields in the $sp$-basis to those in a Cartesian basis,

8 $$\begin{array}{ccccc}\\ E_x({\mathbf{\kappa }}) = & \sin \phi \;E_s({\mathbf{\kappa }}) - \frac{{k_z}}{{k_0}}\cos \phi \;E_p({\mathbf{\kappa }})\\ \\ E_y({\mathbf{\kappa }}) = & - \cos \phi \;E_s({\mathbf{\kappa }}) - \frac{{k_z}}{{k_0}}\sin \phi \;E_p({\mathbf{\kappa }})\\ \\ H_x({\mathbf{\kappa }}) = & \sin \phi \;\frac{{E_p({\mathbf{\kappa }})}}{{Z_0}} + \frac{{k_z}}{{k_0}}\cos \phi \;\frac{{E_s({\mathbf{\kappa }})}}{{Z_0}}{\mathrm{ and}}\\ \\ H_y({\mathbf{\kappa }}) = & - \cos \phi \;\frac{{E_p({\mathbf{\kappa }})}}{{Z_0}} + \frac{{k_z}}{{k_0}}\sin \phi \;\frac{{E_s({\mathbf{\kappa }})}}{{Z_0}}\\ \end{array}$$

These equations are derived in Supplementary Note 5 and can be straightforwardly inverted to find the transformation from a Cartesian to an *sp*-basis.

## Supplementary information

Supplemental material
