# Supplementary material for: A full vectorial mapping of nanophotonic light fields
Source: Light Sci Appl. 2019 Mar 6;8:28. doi: 10.1038/s41377-019-0124-3 (PMC6403231; doi:10.1038/s41377-019-0124-3)
Supplement: Supplementary file 1 — Supplemental material [file 41377_2019_124_MOESM1_ESM.docx]

Supplementary information for:

***A full vectorial mapping of nanophotonic light fields***

Boris le Feber^1,2^, John. E. Sipe^3^, Matthias Wulf^2,4^, L. Kuipers^2,5^ & N. Rotenberg^2,6^

^1^Optical Materials Engineering Laboratory, ETH Zürich, 8092 Zurich, Switzerland

^2^Center for Nanophotonics, AMOLF, Science Park 104, 1098 XG Amsterdam, The Netherlands

^3^Institute for Optical Sciences, University of Toronto, 60 St. George Street, Ontario M5S 1A7,

Canada

^4^Institute of Science and Technology Austria, Am Campus 1, 3400 Klosterneuburg, Austria

^5^Kavli Institute of Nanoscience, Department of Quantum Nanoscience, Delft University of Technology, Lorentzweg 1, 2628 CJ Delft, The Netherlands

^6^Niels Bohr Institute, University of Copenhagen, Blegdamsvej 17, DK-2100 Copenhagen, Denmark

**Supplementary Note 1: Phase- and polarization-resolved NSOM**

***Phase-resolved NSOM***

Access to the optical phase can be gained by incorporating the near-field scanning optical microscope (NSOM) in an interferometric detection scheme, as sketched in Fig. S1. Specifically, we use a heterodyne detection scheme, in which the light from the probe interferes with the frequency shifted reference radiation from the reference branch; see Fig. S1. The frequency of the reference branch is shifted by 40 kHz using two acousto-optic modulators (AOMs); see Fig. S1. By analyzing the beating signal measured on the diodes on two lock-in amplifiers, we gain access to the optical phase of the signal branch.

***Polarization-resolved NSOM***

To measure the polarization of the light emitted from the probe tip, we use a polarizing beam splitter, marked by PBS in Fig. S1, which ensures that light polarized along *x* and *y* contributes to the signals *L_x_* and *L_y_*, respectively. However, to be able to relate light emitted with *x* and *y* polarizations near the sample (indicated in Fig. S1) to *x*- and *y*-polarized light (now in the lab frame) at the detectors, we need to correct for birefringence in the fibers after the probe.

As a result of this birefringence, linear *x*- or *y*-polarized radiation from the probe will typically become elliptically polarized upon transmission through the fiber. To project these elliptical polarizations back onto the *x*- and *y*-orientations above the sample, we employ the quarter- and half-wave plates sketched in Fig. S1. First, after the fiber we use the quarter-wave plate (*λ*/4) to project the elliptically polarized light back onto linearly polarized light. Then, we insert the second half-wave plate (*λ/*2_(2)_) to rotate the light such that *x*- and *y*-polarized radiation from the probe contributes to *L_x_* and *L_x_*, respectively. Finally, we use the first half-wave plate (*λ/*2_(1)_) to balance the intensity of the reference branch over the two detectors.

**
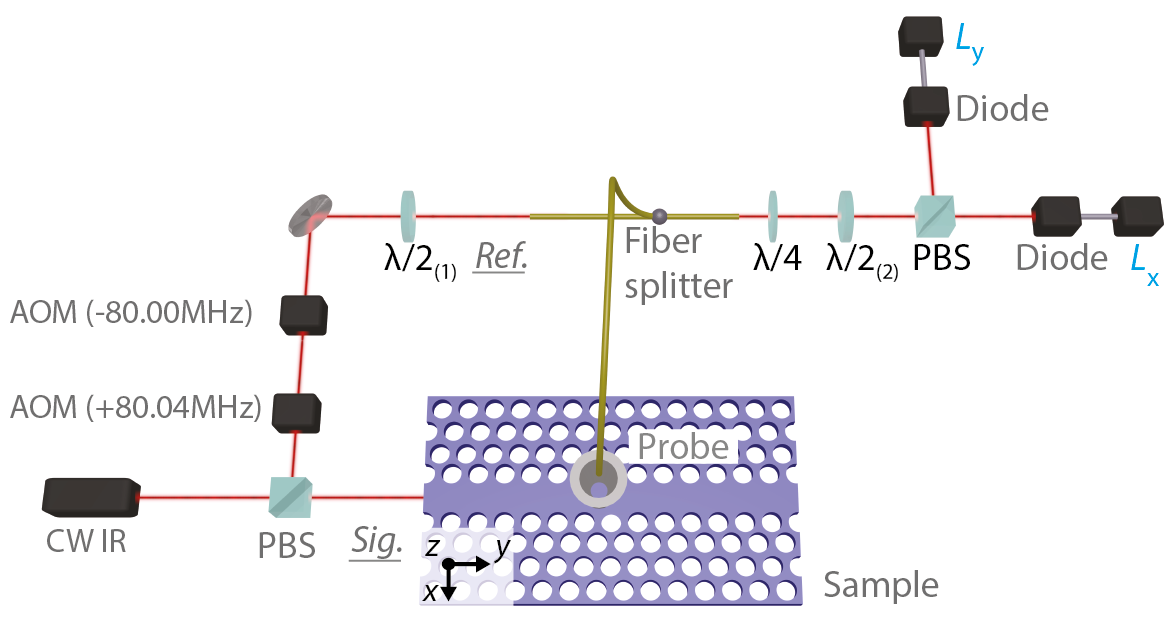
**

**Supplementary Figure S1. Polarization-sensitive NSOM.** Light from a continuous wave infrared laser (CW IR) is split up into a signal (Sig.) and a reference (Ref.) branch. The reference branch is frequency shifted using two AOMs before it is coupled to a fiber (yellow tube). The light in the signal branch is coupled into the sample, from where the light is collected by a near-field probe. Light from the probe propagates through the fiber, where it joins the reference branch. After the fiber splitter the light from the two branches is converted to a free-space beam, which, after passing through the polarizing beam splitter (PBS), is detected on the photodiodes, whose signal is analyzed by the lock-in detectors. This extension adds polarization sensitivity by means of the elements marked with black letters. The elements required for a phase-sensitive NSOM are marked in gray. In blue we indicate the two signals *L_x_* and *L_y_*.

***Height-feedback mechanism***

We use a force feedback loop to keep the probe at the sample when scanning the surface of the photonic crystal while “in contact”. However, if *h* > 20 nm, we can no longer use the force feedback, so we therefore switch to a quadrant-cell-based height-feedback loop. Here, the quadrant-cell that measures the relative position of the probe towards or away from the sample, is fixed to the same frame as the sample and does not move whilst the probe scans the sample. When feeding back on the quadrant cell, we feed back on the distance away from the sample compared to the probe-sample-distance, as measured with the quadrant cell, whilst scanning the surface of the sample [1].

**Supplementary Note 2: Photonic-crystal waveguide mode calculation**

The photonic crystal waveguide is a W1 waveguide, which has a row of missing holes in a 220-nm-thin silicon membrane perforated with a hexagonal pattern of holes (see also Fig. 1a of the main text). In the plane of the slab light is confined to this line by the photonic bandgap of the surrounding holes, and is confined to the silicon slab by total internal reflection. Our waveguide has a hole separation of *a* = 420 nm and a hole radius of *r* = 120 nm = 0.29*a*.

The waveguide eigenmodes **E**(**r**) and **H**(**r**) were calculated with the MIT Photonic-Bands package [2]. We use a supercell of dimensions *a* × 11*a*√3 × 10*h*, where *a* is the lattice constant of the photonic crystal and *h* is the thickness of the silicon slab. This supercell is sufficiently large to avoid interactions between neighboring supercells. The calculations were performed with a grid size of *a*/16, which ensures convergence of the eigenvalues to better than 0.1%. The refractive index of silicon used was modelled to be 3.48, which is suitable for wavelengths around 1570 nm.

**Supplementary Note 3: Number of unknowns**

According to Eq. 1 (main text), in real space, the expected measurement for each probe position **R** can be calculated via the overlap integral of the experimental and reciprocal fields over the complete surface **S**. Here, the experimental fields at each position of the surface are unknown. Therefore, in real space, Eq. 1 contains an infinite number of unknowns for each, individual probe position (**R**_tip_). In practice, the actual number of unknowns in this equation corresponds to 4*m*, where *m* is the number of **R***_tip_* positions that we consider: basically, each component of the four in-plane fields in Eq. 1 ($\mathbf{E}_{x,y}^{e}$ and $\mathbf{H}_{x,y}^{e}$) at each position. In total, we only have 2*m* equations – one for *L_x_* and one for *L_y_* at each position – to deal with these 4*m* unknowns. Crucially, the equations are all interdependent since the signal at one point depends on the fields at all points, meaning that they all must be solved simultaneously. Computationally, this requires the inversion of a 2*m*X2*m* matrix.

Turning to reciprocal space (Eq. 3), we see that there are two equations and four unknowns for each plane wave. Although a reconstruction of the image requires solving Eq. 3 for all plane waves, the benefit of the k-space approach is that these equations are decoupled. That is, we only invert 2X2 matrices (Eq. 5), and we can gain insight to how the signal from each individual plane wave affects the measured signal. Importantly, as is the case with the real-space approach, we must still double the number of equations to match the number of unknowns, so that they can be solved. As we show in the main text, when working in reciprocal space, this can be done by specifying the direction in which the waves are propagating.

**Supplementary Note 4: The optical reciprocity theorem**

***The reciprocity integral***

We begin with the Maxwell equations

$$\begin{matrix} \nabla\times\mathbf{H}\left(\mathbf{r}\right)+i\omega\mathbf{D}\left(\mathbf{r}\right)=\mathbf{J}\left(\mathbf{r}\right), \\ \nabla\cdot\mathbf{D}\left(\mathbf{r}\right)=\rho\left(\mathbf{r}\right), \\ \nabla\times\mathbf{E}\left(\mathbf{r}\right)-i\omega\mathbf{B}\left(\mathbf{r}\right)=0, \\ \nabla\cdot\mathbf{B}\left(\mathbf{r}\right)=0, \end{matrix}$$

where all our fields are stationary,

$$f(\mathbf{r},t)=f(\mathbf{r})e^{-i\omega t}+c.c.,$$

and we consider a position and frequency dependent dielectric constant $\varepsilon(\mathbf{r},\omega)$,

$$\begin{matrix} \mathbf{D}\left(\mathbf{r}\right)=\varepsilon_{0}\varepsilon(\mathbf{r},\omega)\mathbf{E}(\mathbf{r}), \\ \mathbf{B}(\mathbf{r})=\mu_{0}\mathbf{H}(\mathbf{r}). \end{matrix}$$

Note that for stationary fields the divergence conditions in follow immediately from the curl conditions and charge conservation.

We consider two solutions to equation corresponding to two sets of charge and current densities,

$$\begin{matrix} \nabla\times\mathbf{H}^{1}\left(\mathbf{r}\right)+i\omega\mathbf{D}^{1}\left(\mathbf{r}\right)=\mathbf{J}^{1}\left(\mathbf{r}\right), \\ \nabla\cdot\mathbf{D}^{1}\left(\mathbf{r}\right)=\rho^{1}\left(\mathbf{r}\right), \\ \nabla\times\mathbf{E}^{1}\left(\mathbf{r}\right)-i\omega\mathbf{B}^{1}\left(\mathbf{r}\right)=0, \\ \nabla\cdot\mathbf{B}^{1}\left(\mathbf{r}\right)=0, \end{matrix}$$

and

$$\begin{matrix} \nabla\times\mathbf{H}^{2}\left(\mathbf{r}\right)+i\omega\mathbf{D}^{2}\left(\mathbf{r}\right)=\mathbf{J}^{2}\left(\mathbf{r}\right), \\ \nabla\cdot\mathbf{D}^{2}\left(\mathbf{r}\right)=\rho^{2}\left(\mathbf{r}\right), \\ \nabla\times\mathbf{E}^{2}\left(\mathbf{r}\right)-i\omega\mathbf{B}^{2}\left(\mathbf{r}\right)=0, \\ \nabla\cdot\mathbf{B}^{2}\left(\mathbf{r}\right)=0. \end{matrix}$$

Then using

$$\nabla\cdot\left(\mathbf{X}\times\mathbf{Y}\right)=\mathbf{Y}\cdot\left(\nabla\times\mathbf{X}\right)-\mathbf{X}\cdot\left(\nabla\times\mathbf{Y}\right),$$

we have

$$\begin{matrix} \nabla\cdot\left(\mathbf{E}^{1}\times\mathbf{H}^{2}\right)=\mathbf{H}^{2}\cdot\left(\nabla\times\mathbf{E}^{1}\right)-\mathbf{E}^{1}\cdot\left(\nabla\times\mathbf{H}^{2}\right) \\ =i\omega\mathbf{H}^{2}\cdot\mathbf{B}^{1}+i\omega\mathbf{E}^{1}\cdot\mathbf{D}^{2}-\mathbf{E}^{1}\cdot\mathbf{J}^{2}, \end{matrix}$$

and

$$\begin{matrix} \nabla\cdot\left(\mathbf{E}^{2}\times\mathbf{H}^{1}\right)=\mathbf{H}^{2}\cdot\left(\nabla\times\mathbf{E}^{2}\right)-\mathbf{E}^{2}\cdot\left(\nabla\times\mathbf{H}^{1}\right) \\ =i\omega\mathbf{H}^{1}\cdot\mathbf{B}^{2}+i\omega\mathbf{E}^{2}\cdot\mathbf{D}^{1}-\mathbf{E}^{2}\cdot\mathbf{J}^{1}. \end{matrix}$$

Since the two solutions and are at the same frequency we have

$$\mathbf{E}^{1}\left(\mathbf{r}\right)\cdot\mathbf{D}^{2}\left(\mathbf{r}\right)=\mathbf{E}^{1}\left(\mathbf{r}\right)\cdot\mathbf{E}^{2}\left(\mathbf{r}\right)\varepsilon_{0}\varepsilon(\mathbf{r},\omega)=\mathbf{D}^{1}\left(\mathbf{r}\right)\cdot\mathbf{E}^{2}\left(\mathbf{r}\right)$$

and of course

$$\mathbf{H}^{2}\left(\mathbf{r}\right)\cdot\mathbf{B}^{1}\left(\mathbf{r}\right)=\mathbf{H}^{2}\left(\mathbf{r}\right)\cdot\mathbf{H}^{1}\left(\mathbf{r}\right)\mu_{0}=\mathbf{B}^{2}\left(\mathbf{r}\right)\cdot\mathbf{H}^{1}\left(\mathbf{r}\right),$$

so we find

$$\nabla\cdot\left(\mathbf{E}^{1}\times\mathbf{H}^{2}-\mathbf{E}^{2}\times\mathbf{H}^{1}\right)=-\mathbf{E}^{1}\cdot\mathbf{J}^{2}+\mathbf{E}^{2}\cdot\mathbf{J}^{1}$$

or, moving to integral form,

$$\int_{S} \left(\mathbf{E}^{1}\times\mathbf{H}^{2}-\mathbf{E}^{2}\times\mathbf{H}^{1}\right)\cdot d\mathbf{s}=\int_{V} \left(-\mathbf{E}^{1}\cdot\mathbf{J}^{2}+\mathbf{E}^{2}\cdot\mathbf{J}^{1}\right)dv$$

***The scenario of interest***

We now look at our scenario of interest, shown in Fig. S2. We imagine some photonic-crystal structure – or more generally any sample – excited by a charge-current distribution $\left(\rho^{1}(\mathbf{r}),\mathbf{J}^{1}(\mathbf{r})\right)$ located to the right of the structure and below the plane of interest *z_plane_*, which lies in vacuum above the structure. We also imagine an observation structure – tip and fiber in our experiment – that is indicated by a cylinder in Fig. S2 and channels light from the neighborhood of *z_plane_* to the neighborhood of *z_obs_*, the location of an observation plane. Its position in the *xy*-plane is specified by$\mathbf{R}_{tip}=(x_{tip},y_{tip})$. In the plane of interest the electromagnetic field resulting from the driving source is $(\mathbf{E}^{1}(\mathbf{R},\mathbf{R}_{tip},z_{plane}), \mathbf{H}^{1}(\mathbf{R},\mathbf{R}_{tip},z_{plane}))$, where the dependence on $\mathbf{R}_{tip}$ reminds us that in general the field will be modified by the observation structure and its position. The electric field reaching the observation plane through the observation structure is $\mathbf{E}^{1}(\mathbf{R}_{tip},z_{obs})$. This is the quantity we shall detect.


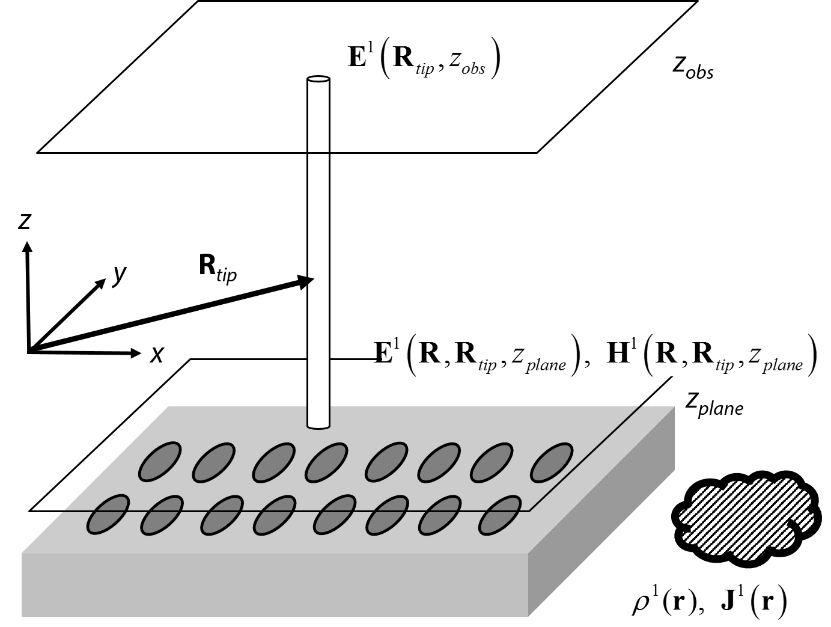


**Supplementary Figure S2: The scenario of interest.** The grey block and grey cloud indicate the photonic crystal (or more generally any sample) and charge current distribution, respectively. The observation structure (tip and fiber) are sketched as a vertical cylinder that is capped at the bottom and top by the observation and detection planes, respectively.

We describe the detection occurring by considering the overlap of this field with a second charge current distribution$(\rho^{2}(\mathbf{r}),\mathbf{J}^{2}(\mathbf{r}))$, which very generally we imagine in the neighborhood of$(\mathbf{R}_{tip},z_{obs})$, see Fig. S3. The fields that this charge distribution generates in the plane of interest are denoted by$(\mathbf{E}^{2}(\mathbf{R},\mathbf{R}_{tip},z_{plane}), \mathbf{H}^{2}(\mathbf{R},\mathbf{R}_{tip},z_{plane}))$.

Now we construct a volume of interest as follows: Imagine a large circle in the plane *z_plane_*, and cap it with a hemisphere. Then imagine increasing the radius of this circle to infinity, enlarging the hemisphere with it.


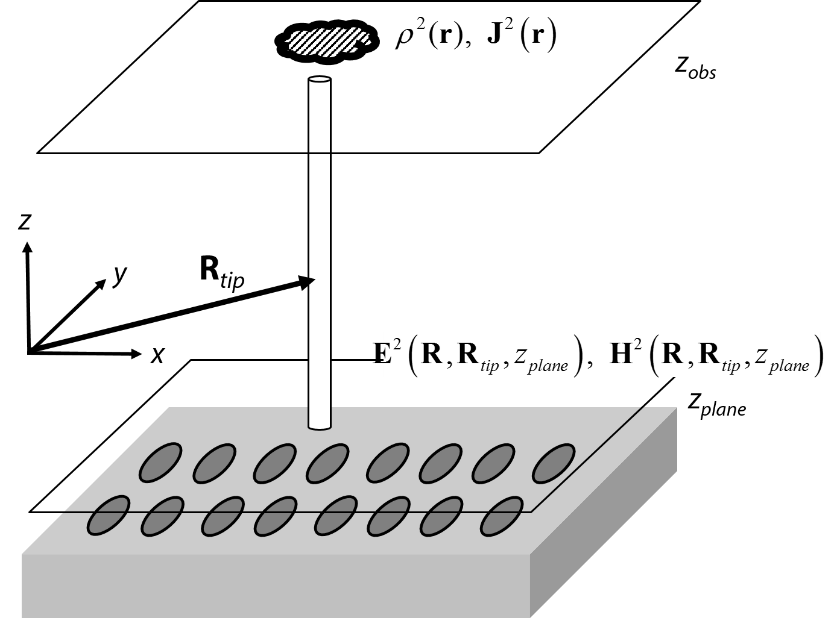


**Supplementary Figure S3: The reciprocal fields.** The grey block and grey cloud indicate the sample and reciprocal charge current distribution, respectively. The tip and fiber are sketched as a vertical cylinder, that is capped in the bottom and top by the observation and detection planes, respectively.

Consider first the surface integral appearing in over the hemisphere. The fields from both the first and the second charge-current distributions are responsible for generating the fields that appear, but as the radius of the hemisphere approaches infinity the Poynting-vector-like terms each drop off as$(radius)^{-2}$, and the difference will drop off faster. Thus only the integral over the plane at $z_{plane}$ (where$d\mathbf{s}=-\hat{\mathbf{z}} dxdy$) will contribute. With respect to the volume integral, only the second charge-current distribution $(\rho^{2}(\mathbf{r}), \mathbf{J}^{2}(\mathbf{r}))$ lies in the volume, so only it will contribute. Thus will simplify here to

$$\begin{matrix} =\int\left(\mathbf{E}^{1}(\mathbf{r},\mathbf{R}_{tip})\cdot\mathbf{J}^{2}\left(\mathbf{r}\right)\right)dv \\ =\int\left(\mathbf{E}^{1}(\mathbf{R},\mathbf{R}_{tip},z_{plane})\times\mathbf{H}^{2}(\mathbf{R},\mathbf{R}_{tip},z_{plane})-\mathbf{E}^{2}(\mathbf{R},\mathbf{R}_{tip},z_{plane})\times\mathbf{H}^{1}(\mathbf{R},\mathbf{R}_{tip},z_{plane})\right)\cdot\hat{\mathbf{z}} dxdy \end{matrix}$$

In practice we will employ a $\mathbf{J}^{2}(\mathbf{r})$ below that also depends on the position $\mathbf{R}_{tip}$ of the observation structure.


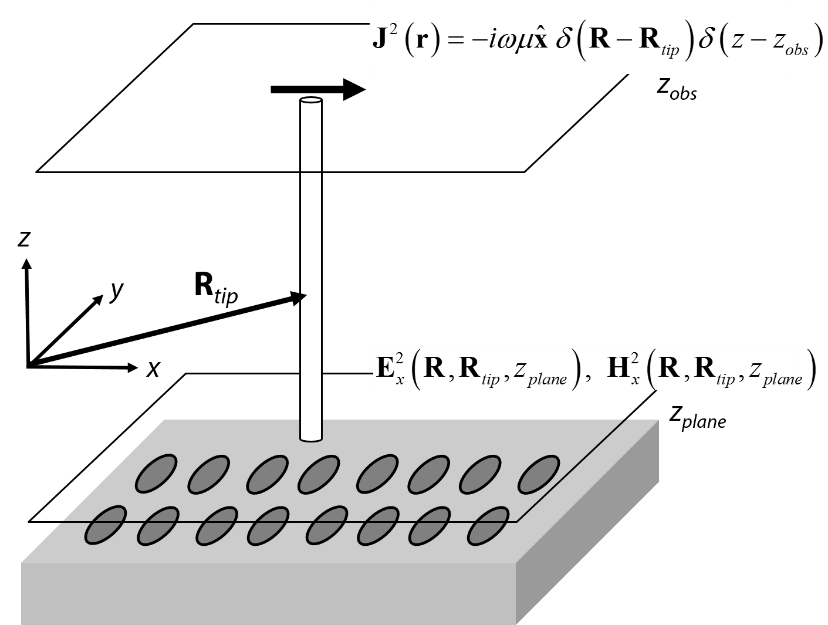


**Supplementary Figure S4: One of two dipole-moment directions considered.** The grey block indicates the Sample and the black arrow show the reciprocal dipole. The tip and fiber are sketched as a vertical cylinder, that is capped in the bottom and top by the observation and detection planes, respectively.

We now consider two special current densities$\mathbf{J}^{2}(\mathbf{r})$. Each corresponds to a point dipole $\mathbf{\mu}$ located at$(\mathbf{R}_{tip},z_{obs})$; in one case we take the dipole oriented along the $\hat{\mathbf{x}}$ direction, and in the other we take it oriented along the $\hat{\mathbf{y}}$ direction. The first case is illustrated in Fig. 4; the current density is

$$\mathbf{J}^{2}(\mathbf{r})=-i\omega\mu\hat{\mathbf{x}}\delta(\mathbf{R}-\mathbf{R}_{tip})\delta(z-z_{obs}).$$

For this orientation of the dipole, we denote the fields $(\mathbf{E}^{2}(\mathbf{R},\mathbf{R}_{tip},z_{plane}),\mathbf{H}^{2}(\mathbf{R},\mathbf{R}_{tip},z_{plane}))$ that result at $z=z_{plane}$ by $(\mathbf{E}_{x}^{2}(\mathbf{R},\mathbf{R}_{tip},z_{plane}),\mathbf{H}_{x}^{2}(\mathbf{R},\mathbf{R}_{tip},z_{plane}))$, and becomes

$$\begin{matrix} =-i\omega\mu\left(\hat{\mathbf{x}}\cdot\mathbf{E}^{1}(\mathbf{R}_{tip},z_{obs})\right) \\ =\int\left(\mathbf{E}^{1}(\mathbf{R},\mathbf{R}_{tip},z_{plane})\times\mathbf{H}_{x}^{2}(\mathbf{R},\mathbf{R}_{tip},z_{plane})-\mathbf{E}_{x}^{2}(\mathbf{R},\mathbf{R}_{tip},z_{plane})\times\mathbf{H}^{1}(\mathbf{R},\mathbf{R}_{tip},z_{plane})\right)\cdot\hat{\mathbf{z}} dxdy, \end{matrix}$$

and a similar expression can be written down if we take the dipole $\mathbf{\mu}$ to be oriented in the $\hat{\mathbf{y}}$ direction. If we put

where $i=x,y$, we then have

$$\begin{matrix} =L_{i}(\mathbf{R}_{tip},z_{obs}) \\ =\int\left(\mathbf{E}^{1}(\mathbf{R},\mathbf{R}_{tip},z_{plane})\times\mathbf{H}_{i}^{2}(\mathbf{R},\mathbf{R}_{tip},z_{plane})-\mathbf{E}_{i}^{2}(\mathbf{R},\mathbf{R}_{tip},z_{plane})\times\mathbf{H}^{1}(\mathbf{R},\mathbf{R}_{tip},z_{plane})\right)\cdot\hat{\mathbf{z}} dxdy \end{matrix}$$

So far everything is still exact. We assume that the quantities $L_{i}(\mathbf{R}_{tip},z_{obs})$ are measurable; that is, both the amplitude and the phase of the complex quantities can be detected in the laboratory.

***The approximations***

We now introduce some approximations in the form of . First, we assume that the fields $(\mathbf{E}^{1}(\mathbf{R},\mathbf{R}_{tip},z_{plane}), \mathbf{H}^{1}(\mathbf{R},\mathbf{R}_{tip},z_{plane}))$ are to good approximation unaffected by the presence of the observation structure. That is, at $z=z_{plane}$ we can calculate the fields $(\mathbf{E}^{1}(\mathbf{R},\mathbf{R}_{tip},z_{plane}), \mathbf{H}^{1}(\mathbf{R},\mathbf{R}_{tip},z_{plane}))$ as if the observation structure were not present (see Fig. S5).


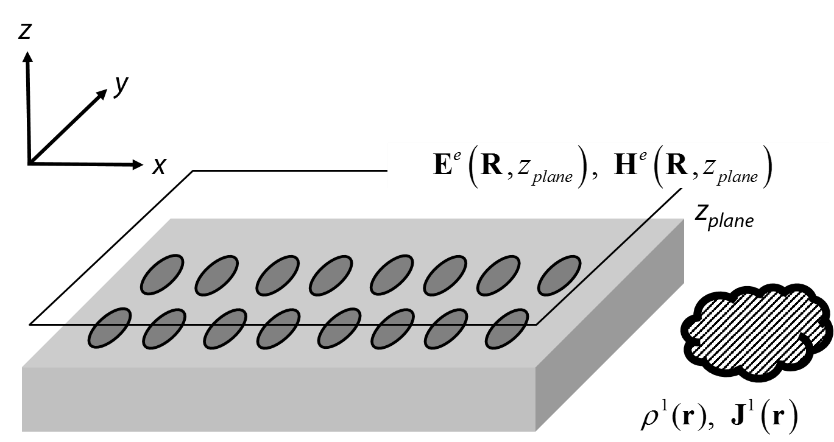


**Supplementary Figure S5: The approximation that the photonic-crystal fields at the plane of interest are unaffected by the observation structure.** The grey block and grey cloud indicate the sample and charge current distribution, respectively. The tip is not sketched, to illustrate that we assume that it does not affect the experimental fields.

Within this approximation the dependence of $(\mathbf{E}^{1}(\mathbf{R},\mathbf{R}_{tip},z_{plane}), \mathbf{H}^{1}(\mathbf{R},\mathbf{R}_{tip},z_{plane}))$ on $\mathbf{R}_{tip}$ vanishes, and we put $(\mathbf{E}^{1}(\mathbf{R},\mathbf{R}_{tip},z_{plane}), \mathbf{H}^{1}(\mathbf{R},\mathbf{R}_{tip},z_{plane}))\to(\mathbf{E}^{e}(\mathbf{R},z_{plane}), \mathbf{H}^{e}(\mathbf{R},z_{plane}))$ in , where the superscript $e$ indicates the fields in the experimental situation discussed in the main text.

Second, we assume that the fields $(\mathbf{E}_{i}^{2}(\mathbf{R},\mathbf{R}_{tip},z_{plane}), \mathbf{H}_{i}^{2}(\mathbf{R},\mathbf{R}_{tip},z_{plane}))$ are to good approximation unaffected by the presence of the sample. Thus, they can be evaluated as if the photonic crystal were not present; see Fig. S6.


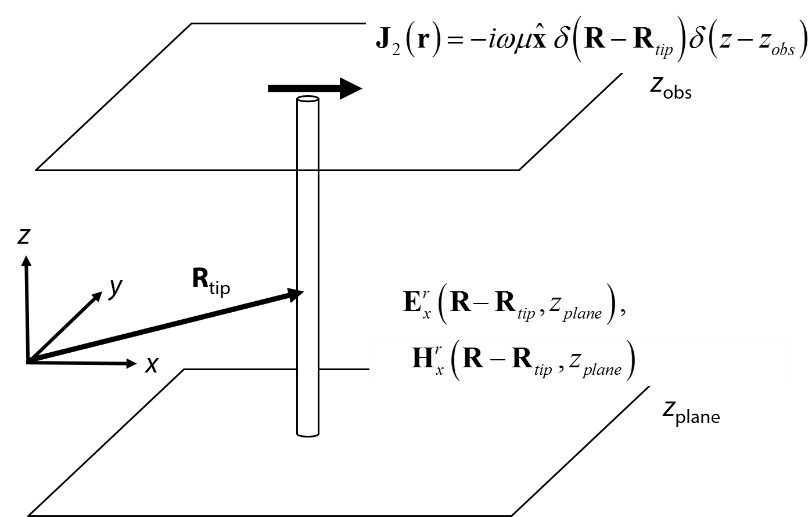


**Supplementary Figure S6: The approximation that the reciprocal fields at the plane of interest are unaffected by the sample.** The black arrow show the dipole current that sets up the reciprocal fields, which we assume are unperturbed by the sample.

Then, although these fields at *z* = *z_plane_* do still depend on **R***_tip_*, they will only depend on the difference **R**-**R***_tip_*, and we can write

$(\mathbf{E}_{i}^{2}(\mathbf{R},\mathbf{R}_{tip},z_{plane}), \mathbf{H}_{i}^{2}(\mathbf{R},\mathbf{R}_{tip},z_{plane}))\to(\mathbf{E}_{i}^{r}(\mathbf{R}-\mathbf{R}_{tip},z_{plane}), \mathbf{H}_{i}^{r}(\mathbf{R}-\mathbf{R}_{tip},z_{plane}))$,

where the superscript *r* indicates the fields in the reciprocal situation discussed in the main text. Using these approximations in we have

$$\begin{matrix} =L_{i}(\mathbf{R}_{tip},z_{obs}) \\ =\int_{S} \left(\mathbf{E}^{e}(\mathbf{R},z_{plane})\times\mathbf{H}_{i}^{r}(\mathbf{R}-\mathbf{R}_{tip},z_{plane})-\mathbf{E}_{i}^{r}(\mathbf{R}-\mathbf{R}_{tip},z_{plane})\times\mathbf{H}^{e}(\mathbf{R},z_{plane})\right)\cdot\hat{\mathbf{z}} dxdy. \end{matrix}$$

Please note that, for clarity and brevity, we have omitted the dependence on *z_obs_* and *z_plane_* in the main text. The former affects only the overall amplitude scaling of the signals. Throughout our manuscript we omit this dependence and only compare the relative strengths between field components and signals. The latter, *z_plane_*, is fixed to 10 nm below the probe apex throughout this work.

**Supplementary Note 5: Basis Conversion**

***The move to*** $\kappa$ ***space***

Next we Fourier decompose in the *xy*-plane, writing

$$\mathbf{E}^{e}(\mathbf{R},z)=\int\frac{d\mathbf{\kappa}}{(2\pi)^{2}}\mathbf{E}^{e}(\mathbf{\kappa},z)e^{i\mathbf{\kappa}\cdot\mathbf{R}},$$

etc., where $d\mathbf{\kappa}=d\kappa_{x}d\kappa_{y}$. Then, we can write

where

The integral over $\mathbf{R}=(x,y)$ vanishes unless $\mathbf{\kappa}'=-\mathbf{\kappa}$ , so from (S8) we have

$$\begin{matrix} =L_{i}(\mathbf{R}_{tip},z_{obs}) \\ =\int\frac{d\mathbf{\kappa}}{(2\pi)^{2}}\left(\mathbf{E}^{e}(\mathbf{\kappa},z_{plane})\times\mathbf{H}_{i}^{r}(-\mathbf{\kappa},z_{plane})-\mathbf{E}_{i}^{r}(-\mathbf{\kappa},z_{plane})\times\mathbf{H}^{e}(\mathbf{\kappa},z_{plane})\right)\cdot\hat{\mathbf{z}} e^{i\mathbf{\kappa}\cdot\mathbf{R}_{tip}}. \end{matrix}$$

Now putting

$$L_{i}(\mathbf{R}_{tip},z_{obs})=\int\frac{d\mathbf{\kappa}}{(2\pi)^{2}}L_{i}(\mathbf{\kappa},z_{obs})e^{i\mathbf{\kappa}\cdot\mathbf{R}_{tip}},$$

we see that

$$L_{i}(\mathbf{\kappa},z_{obs})=\left(\mathbf{E}^{e}(\mathbf{\kappa},z_{plane})\times\mathbf{H}_{i}^{r}(-\mathbf{\kappa},z_{plane})-\mathbf{E}_{i}^{r}(-\mathbf{\kappa},z_{plane})\times\mathbf{H}^{e}(\mathbf{\kappa},z_{plane})\right)\cdot\hat{\mathbf{z}}$$

or, writing out the components,

$$\begin{matrix} L_{i}(\mathbf{\kappa},z_{obs})=E_{x}^{e}(\mathbf{\kappa},z_{plane})H_{i,y}^{r}(-\mathbf{\kappa},z_{plane})-E_{y}^{e}(\mathbf{\kappa},z_{plane})H_{i,x}^{r}(-\mathbf{\kappa},z_{plane}) \\ -E_{i,x}^{r}(-\mathbf{\kappa},z_{plane})H_{y}^{e}(\mathbf{\kappa},z_{plane})+E_{i,y}^{r}(-\mathbf{\kappa},z_{plane})H_{x}^{e}(\mathbf{\kappa},z_{plane}). \end{matrix}$$

***Fields in vacuum***

Now let us consider the nature of an electromagnetic in the neighborhood of $z$ where, within that neighborhood, there are no sources of any sort. Then in the neighborhood of $z$ the Maxwell equations reduce to

$$\begin{matrix} \nabla\times\mathbf{H}+i\omega\mathbf{D}=0, \\ \nabla\cdot\mathbf{E}=0, \\ \nabla\times\mathbf{E}-i\omega\mathbf{B}=0, \\ \nabla\cdot\mathbf{B}=0, \end{matrix}$$

with , $\mathbf{B}=\mu_{0}\mathbf{H}$. Fourier transforming the fields in the *xy-*plane,

$$\begin{matrix} \mathbf{E}(\mathbf{r})=\mathbf{E}\left(\mathbf{R},z\right)=\int\frac{d\mathbf{\kappa}}{(2\pi)^{2}}\mathbf{E}(\mathbf{\kappa},z)e^{i\mathbf{\kappa}\cdot\mathbf{R}}, \\ \mathbf{H}(\mathbf{r})=\mathbf{H}\left(\mathbf{R},z\right)=\int\frac{d\mathbf{\kappa}}{(2\pi)^{2}}\mathbf{H}(\mathbf{\kappa},z)e^{i\mathbf{\kappa}\cdot\mathbf{R}}, \end{matrix}$$

the terms $\mathbf{E}(\mathbf{\kappa},z)$ and $\mathbf{H}(\mathbf{\kappa},z)$ are composed of upward propagating (or evanescent) waves and downward propagating (or evanescent) waves. That is, we have

$$\begin{matrix} \mathbf{E}(\mathbf{\kappa},z)=\left(\hat{\mathbf{s}}E_{s+}(\mathbf{\kappa})+\hat{\mathbf{p}}_{+}E_{p+}(\mathbf{\kappa})\right)e^{ik_{z}z} \\ +\left(\hat{\mathbf{s}}E_{s-}(\mathbf{\kappa})+\hat{\mathbf{p}}_{-}E_{p-}(\mathbf{\kappa})\right)e^{-ik_{z}z} \end{matrix}$$

where

$$k_{z}=\sqrt{k_{0}^{2}-\kappa^{2}},$$

with $k_{0}=\omega/c$; since here the argument of the square root is always real, we take $k_{z}$ to be either a positive real number or a positive imaginary number. This guarantees that the + fields are associated with upward propagating (or evanescent) fields and the – fields are associated with downward propagating (or evanescent) fields. And here

$$\hat{\mathbf{s}}=\hat{\mathbf{\kappa}}\times\hat{\mathbf{z}},$$

and

$$\hat{\mathbf{p}}_{\pm}=\frac{\kappa\hat{\mathbf{z}}\mp k_{z}\hat{\mathbf{\kappa}}}{k_{0}}.$$

For each $\mathbf{\kappa}$ there are four independent quantities in ,$E_{s+}(\mathbf{\kappa})$, $E_{p+}(\mathbf{\kappa})$, $E_{s-}(\mathbf{\kappa})$, and $E_{p-}(\mathbf{\kappa})$. Since from the Maxwell equations we can write the corresponding expression for $\mathbf{H}(\mathbf{\kappa},z)$ as

$$\begin{matrix} \mathbf{H}(\mathbf{\kappa},z)=\frac{\left(\hat{\mathbf{s}}E_{p+}(\mathbf{\kappa})-\hat{\mathbf{p}}_{+}E_{s+}(\mathbf{\kappa})\right)}{Z_{0}}e^{ik_{z}z} \\ +\frac{\left(\hat{\mathbf{s}}E_{p-}(\mathbf{\kappa})-\hat{\mathbf{p}}_{-}E_{s-}(\mathbf{\kappa})\right)}{Z_{0}}e^{-ik_{z}z} \end{matrix}$$

there are no additional independent quantities; we have only upward propagating (or evanescent) waves of *s*- and *p*-polarization type, and downward propagating (or evanescent) waves of *s*- and *p*-polarization type.

**Supplementary Figure S7: Unit vectors**

***The experimental fields***

Because we measure above the sample, we can assume for the experimental fields that we have no amplitudes propagating (or evanescent) in the downward direction. Then from and we have

$$\begin{matrix} \mathbf{E}^{e}(\mathbf{\kappa},z)=\left(\hat{\mathbf{s}}E_{s+}(\mathbf{\kappa})+\hat{\mathbf{p}}_{+}E_{p+}(\mathbf{\kappa})\right)e^{ik_{z}z}, \\ \mathbf{H}^{e}(\mathbf{\kappa},z)=\frac{\left(\hat{\mathbf{s}}E_{p+}(\mathbf{\kappa})-\hat{\mathbf{p}}_{+}E_{s+}(\mathbf{\kappa})\right)}{Z_{0}}e^{ik_{z}z}, \end{matrix}$$

and so

$$\begin{matrix} \mathbf{E}^{e}(\mathbf{\kappa},z_{plane})=\hat{\mathbf{s}}E_{s+}(\mathbf{\kappa},z_{plane})+\hat{\mathbf{p}}_{+}E_{p+}(\mathbf{\kappa},z_{plane}), \\ \mathbf{H}^{e}(\mathbf{\kappa},z_{plane})=\hat{\mathbf{s}}\frac{E_{p+}(\mathbf{\kappa},z_{plane})}{Z_{0}}-\hat{\mathbf{p}}_{+}\frac{E_{s+}(\mathbf{\kappa},z_{plane})}{Z_{0}}, \end{matrix}$$

where

$$E_{s,p+}(\mathbf{\kappa},z)\equiv E_{s,p+}(\mathbf{\kappa})e^{ik_{z}z}.$$

Then using we have

$$\begin{matrix} E_{x}^{e}(\mathbf{\kappa},z_{plane})=\left(\hat{\mathbf{x}}\cdot\hat{\mathbf{s}}\right)E_{s+}(\mathbf{\kappa},z_{plane})-\frac{k_{z}}{k_{0}}\left(\hat{\mathbf{x}}\cdot\hat{\mathbf{\kappa}}\right)E_{p+}(\mathbf{\kappa},z_{plane}), \\ E_{y}^{e}(\mathbf{\kappa},z_{plane})=\left(\hat{\mathbf{y}}\cdot\hat{\mathbf{s}}\right)E_{s+}(\mathbf{\kappa},z_{plane})-\frac{k_{z}}{k_{0}}\left(\hat{\mathbf{y}}\cdot\hat{\mathbf{\kappa}}\right)E_{p+}(\mathbf{\kappa},z_{plane}), \\ H_{x}^{e}(\mathbf{\kappa},z_{plane})=\left(\hat{\mathbf{x}}\cdot\hat{\mathbf{s}}\right)\frac{E_{p+}(\mathbf{\kappa},z_{plane})}{Z_{0}}+\frac{k_{z}}{k_{0}}\left(\hat{\mathbf{x}}\cdot\hat{\mathbf{\kappa}}\right)\frac{E_{s+}(\mathbf{\kappa},z_{plane})}{Z_{0}}, \\ H_{y}^{e}(\mathbf{\kappa},z_{plane})=\left(\hat{\mathbf{y}}\cdot\hat{\mathbf{s}}\right)\frac{E_{p+}(\mathbf{\kappa},z_{plane})}{Z_{0}}+\frac{k_{z}}{k_{0}}\left(\hat{\mathbf{y}}\cdot\hat{\mathbf{\kappa}}\right)\frac{E_{s+}(\mathbf{\kappa},z_{plane})}{Z_{0}}. \end{matrix}$$

We introduce an angle which indicates the direction that $\hat{\mathbf{\kappa}}$ makes from the $\hat{\mathbf{x}}$ axis in the *xy* plane,

see Fig. S7, in terms of which we have

From Eq. S9 we then have

or

$$Z_{0}L_{i}(\mathbf{\kappa},z_{obs})=N_{i,s}(\mathbf{\kappa})E_{s+}(\mathbf{\kappa},z_{plane})+N_{i,p}(\mathbf{\kappa})E_{p+}(\mathbf{\kappa},z_{plane}),$$

where

and

We can write as

$$Z_{0}\left[\begin{matrix} L_{x}(\mathbf{\kappa},z_{obs}) \\ L_{y}(\mathbf{\kappa},z_{obs}) \end{matrix}\right]=\text{N(}\mathbf{\kappa}\text{)}\left[\begin{matrix} E_{s+}(\mathbf{\kappa},z_{plane}) \\ E_{p+}(\mathbf{\kappa},z_{plane}) \end{matrix}\right],$$

where for each $\mathbf{\kappa}$ the $2\times2$ matrix $\text{N(}\mathbf{\kappa})$ is given by

$$\text{N(}\mathbf{\kappa})=\left[\begin{matrix} N_{x,s}(\mathbf{\kappa}) & N_{x,p}(\mathbf{\kappa}) \\ N_{y,s}(\mathbf{\kappa}) & N_{y,p}(\mathbf{\kappa}) \end{matrix}\right],$$

and as long as $\det\text{N(}\mathbf{\kappa})\neq0$ we can invert to give

$$\left[\begin{matrix} E_{s+}(\mathbf{\kappa},z_{plane}) \\ E_{p+}(\mathbf{\kappa},z_{plane}) \end{matrix}\right]=Z_{0}\text{N}^{-1}(\mathbf{\kappa})\left[\begin{matrix} L_{x}(\mathbf{\kappa},z_{obs}) \\ L_{y}(\mathbf{\kappa},z_{obs}) \end{matrix}\right]$$

Once this is determined we can find the electric field and the magnetic field anywhere above the sample by using and ,

$$\begin{matrix} \mathbf{E}^{e}(\mathbf{\kappa},z)=\left(\hat{\mathbf{s}}E_{s+}(\mathbf{\kappa},z_{plane})+\hat{\mathbf{p}}_{+}E_{p+}(\mathbf{\kappa},z_{plane})\right)e^{ik_{z}(z-z_{plane})}, \\ Z_{0}\mathbf{H}^{e}(\mathbf{\kappa},z)=\left(\hat{\mathbf{s}}E_{p+}(\mathbf{\kappa},z_{plane})-\hat{\mathbf{p}}_{+}E_{s+}(\mathbf{\kappa},z_{plane})\right)e^{ik_{z}(z-z_{plane})}, \end{matrix}$$

and so, using ,

$$\begin{matrix} \mathbf{E}^{e}(\mathbf{r})=\int\frac{d\mathbf{\kappa}}{(2\pi)^{2}}\left(\hat{\mathbf{s}}E_{s+}(\mathbf{\kappa},z_{plane})+\hat{\mathbf{p}}_{+}E_{p+}(\mathbf{\kappa},z_{plane})\right)e^{i\mathbf{\kappa}\cdot\mathbf{R}}e^{ik_{z}(z-z_{plane})}, \\ Z_{0}\mathbf{H}^{e}(\mathbf{r})=\int\frac{d\mathbf{\kappa}}{(2\pi)^{2}}\left(\hat{\mathbf{s}}E_{p+}(\mathbf{\kappa},z_{plane})-\hat{\mathbf{p}}_{+}E_{s+}(\mathbf{\kappa},z_{plane})\right)e^{i\mathbf{\kappa}\cdot\mathbf{R}}e^{ik_{z}(z-z_{plane})}. \end{matrix}$$

the Cartesian components can be extracted. Again, please note that for clarity and brevity we have omitted the dependence on $z_{obs}$ and $z_{plane}$ in the main text.

**Supplementary Note 6: Plasmonic nanowire field retrieval**

***Fabricating the plasmonic nanowires***

To couple light to the nanowire we use a metal hole-array and waveguide-taper [3]. The nanowire, waveguide-taper, and hole-array are patterned by electron beam lithography into a bilayer PMMA resist. The Au is evaporated through resistive heating on the patterned sample, followed by liftoff. The hole-array has a pitch of 1 μm and a hole-diameter of roughly 0.5 μm. The nanowire length is approximately 50 μm.

***Plasmonic nanowire mode calculations***

The optical modes in the 130 nm wide and 50 nm thick plasmonic nanowire were calculated with a COMSOL 2D-eigenmode analysis. We use a wavelength of 1550 nm, a refractive index of 1.5 for the BK7, and Johnson and Christy values for the gold [4]. Edges of the nanowire were rounded with a 20 nm radius of curvature. We extract the fields from these calculations along the path shown in Fig. S8.


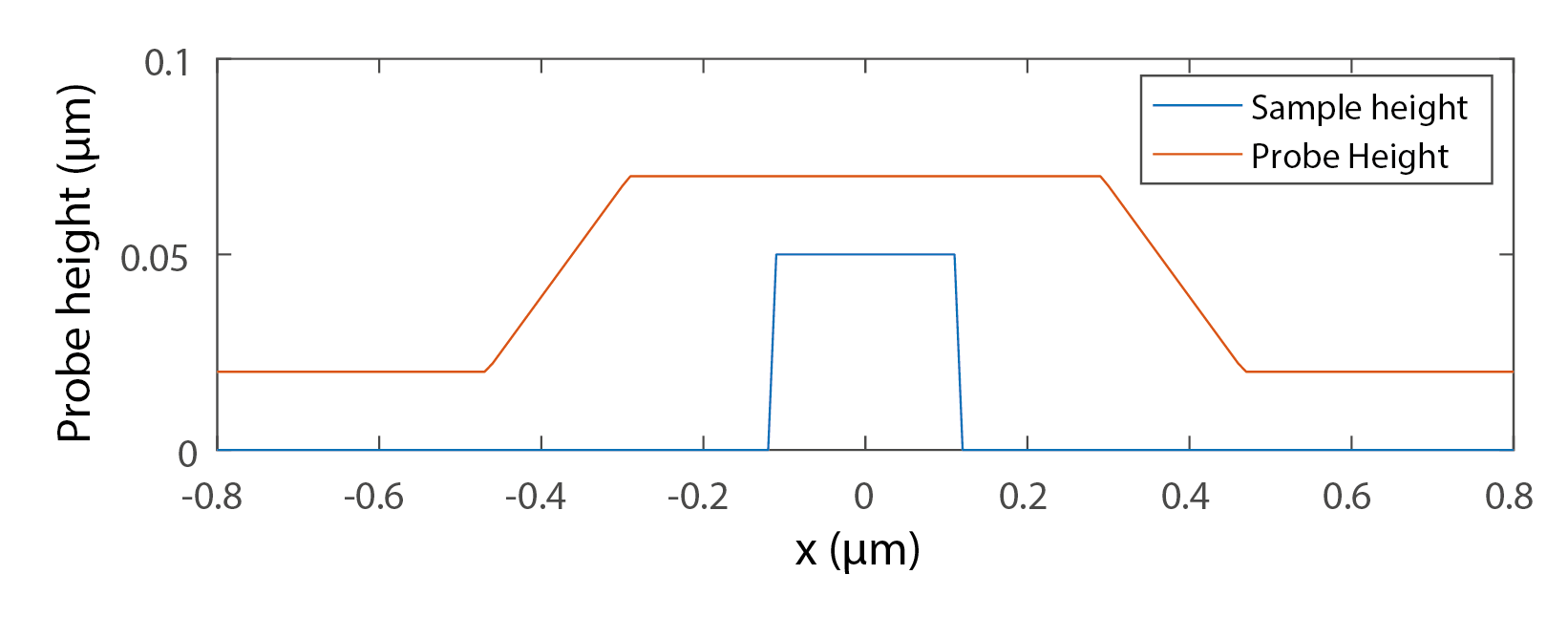


**Supplementary Figure S8: Nanowire scan path.** The blue line indicates the top of the sample in a cross section along *x*. We extracted the data from the simulations of the plasmonic nanowire along the red line.

***Polarization mixing removal***

For the nanowire studies we use the measurements presented in [5]. Those measurements contain a small degree of polarization mixing. Such polarization mixing can arise if the waveplates of the setup are not set completely perfectly, if the tip has a slight asymmetry. Light that is emitted *x*-polarized from the tip, is detected not only on *L_x_* but also on *L_y_* and similarly for light emitted *y*-polarized. Fortunately, we can identify and filter this mixing by using the symmetry properties of the structures’ optical fields. The optical fields of the mode of a nanophotonic structure match the structures’ symmetries [6]. However, the measured fields above the plasmonic nanowire are not symmetric about the center of the waveguide. That is, if we were to assume the center of the waveguide was at *x=*0, *L_y_* would be nearly symmetrical, but *L_x_* would not.

Such a breaking of symmetry is indicative of polarization mixing [6]. Here, we employ the approach we presented earlier in Ref. [6] to remove this mixing. The essence of this removal lies in the symmetries of the fields of a TM mode. For a TM mode, *E_y_*, *E_z_*, and *H_x_* have an even symmetry around the waveguide center, whereas the other components have odd symmetry. As a result, *L_x_* has even symmetry about *x =* 0, whereas that in *L_y_* has odd symmetry. By mirroring both maps around *x* = 0 and adding or subtracting the mirrored to the original maps, we can obtain the even and odd symmetry contributions to both channels.


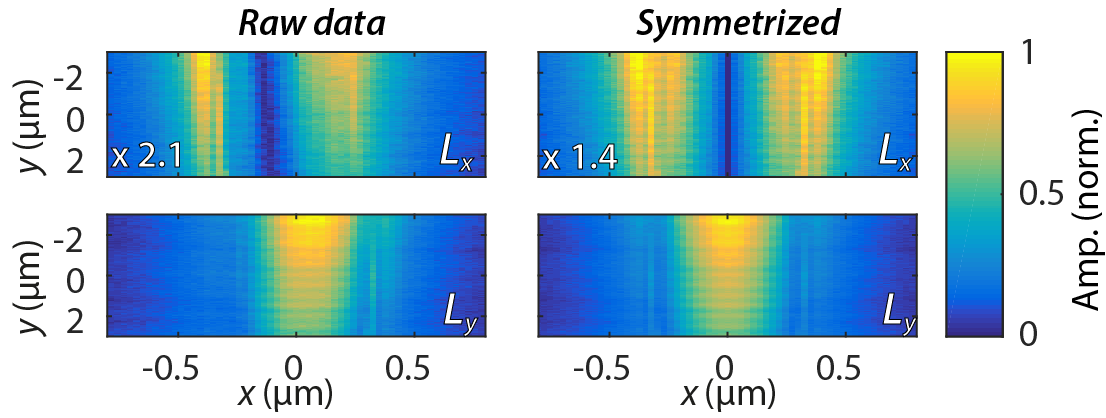


**Supplementary Figure S9: Measured field maps on a plasmonic nanowire.** The left column shows the amplitude of the signal measured on *L_x_* and *L_y_*. The right column show the odd symmetry (top panel) and even symmetry (bottom panel) contributions to *L_x_* and *L_y_*. The color of all maps is scaled to the maximum amplitude (norm.); scaling of *L_x_* relative to *L_y_* is indicated by the multiplication factors in the bottom left of the top panels.

Supplementary Figure S9 shows the results of this approach. The two panels in the left column show the raw measured amplitudes. We symmetrize these panels around the *x* = 0 center of the waveguide. The symmetrized signals are shown in the right panels. As explained in the main text we retrieve the experimental optical fields from these symmetrized fields.

***Field retrieval***

We now insert these symmetrized fields in the deconvolution algorithm to retrieve the maps of the experimental optical fields. These field maps, which are shown in Fig. S10, qualitatively agree with the calculated maps. That is, *E_x_*, and *H_y_* (and *E_y_*, *H_x_*) show a zero (and a maximum) in the center, reminiscent of an odd (and even) symmetry fields. Further, the *H_y_* component we retrieve is more spread out in *x* than *E_x_*, which we also see in our calculated fields. Likewise, in both experiment and theory *H_x_*, is more confined than *E_y_*. Notably, the agreement between experiment and theory is further established by the enhanced side lobes that are visible in both the calculated and retrieved *H_x_* but not in *E_y_*. We also observe that all retrieved fields are slightly less confined than the calculated fields, which we attribute to the finite size of the Fourier filter we used.


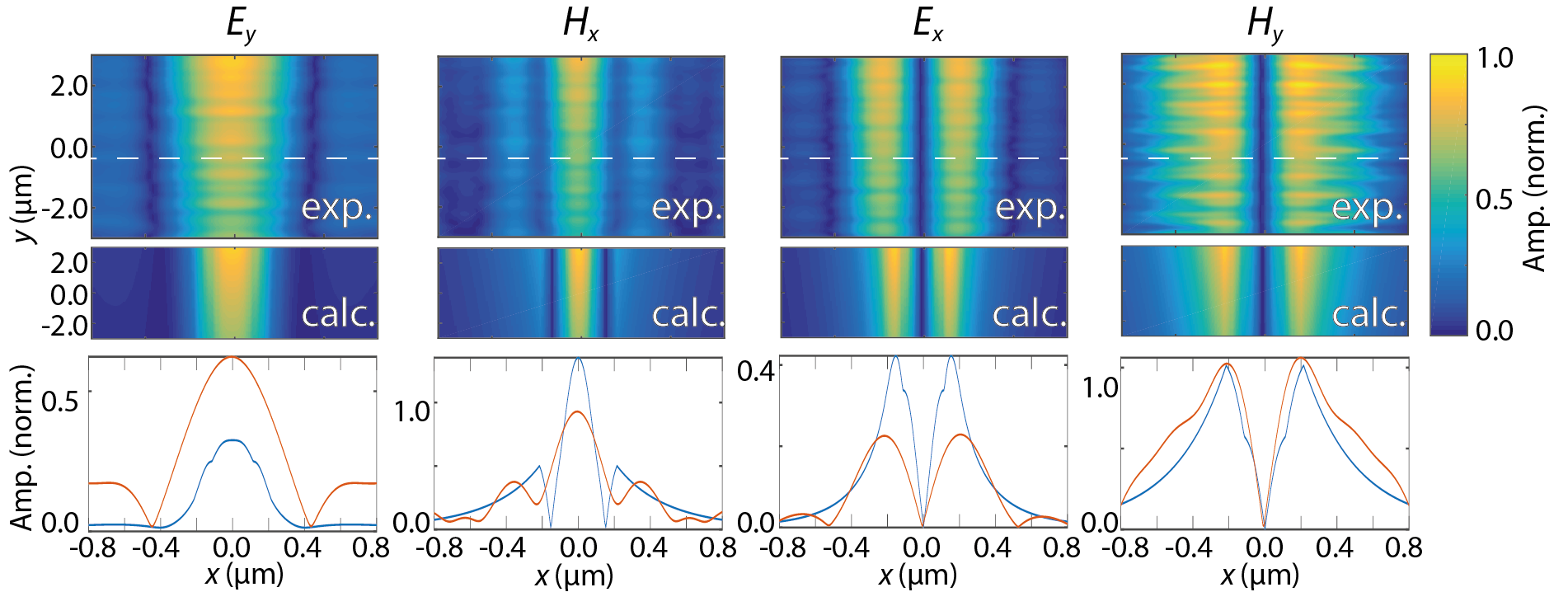


**Supplementary Figure S10: Retrieved nanowire electric and magnetic fields.** Panels show two-dimensional maps and line cuts of the calculated and reconstructed electric and magnetic fields above the plasmonic nanowire. The top (and middle) row of panels show the amplitude of the retrieved and calculated field maps, respectively. Each panel is scaled to its maximum. The bottom row of panels shows line cuts taken along the white dashed lines in the field maps. Red and blue lines correspond to line cuts through the fields reconstructed from the experimental data and calculated fields, respectively.

**Supplementary Note 7: Setting the correct maximum filter**

To select a value for $\kappa_{\text{max}}$ we follow an empirical approach. That is, for $\kappa_{\text{max}}=0$ to $\kappa_{\text{max}}=30k_{0}$, we calculate the mean (of the absolute of the) difference in the electric field amplitude from pixel to pixel in the retrieved distributions. Fig. S11, which depicts the results of this approach, clearly shows a low amount of pixel to pixel noise for $\kappa_{\text{max}}\leq9k_{0}$ (the first plateau), after which this difference rapidly increases by an order of magnitude. Furthermore, when $\kappa_{\text{max}}\leq2k_{0}$the pixel to pixel noise falls off, but those filters are practically not relevant because they will miss essential features of the near field. Combined, these observations suggest that filter $\kappa_{\text{max}}$ values on the first plateau are most suitable.


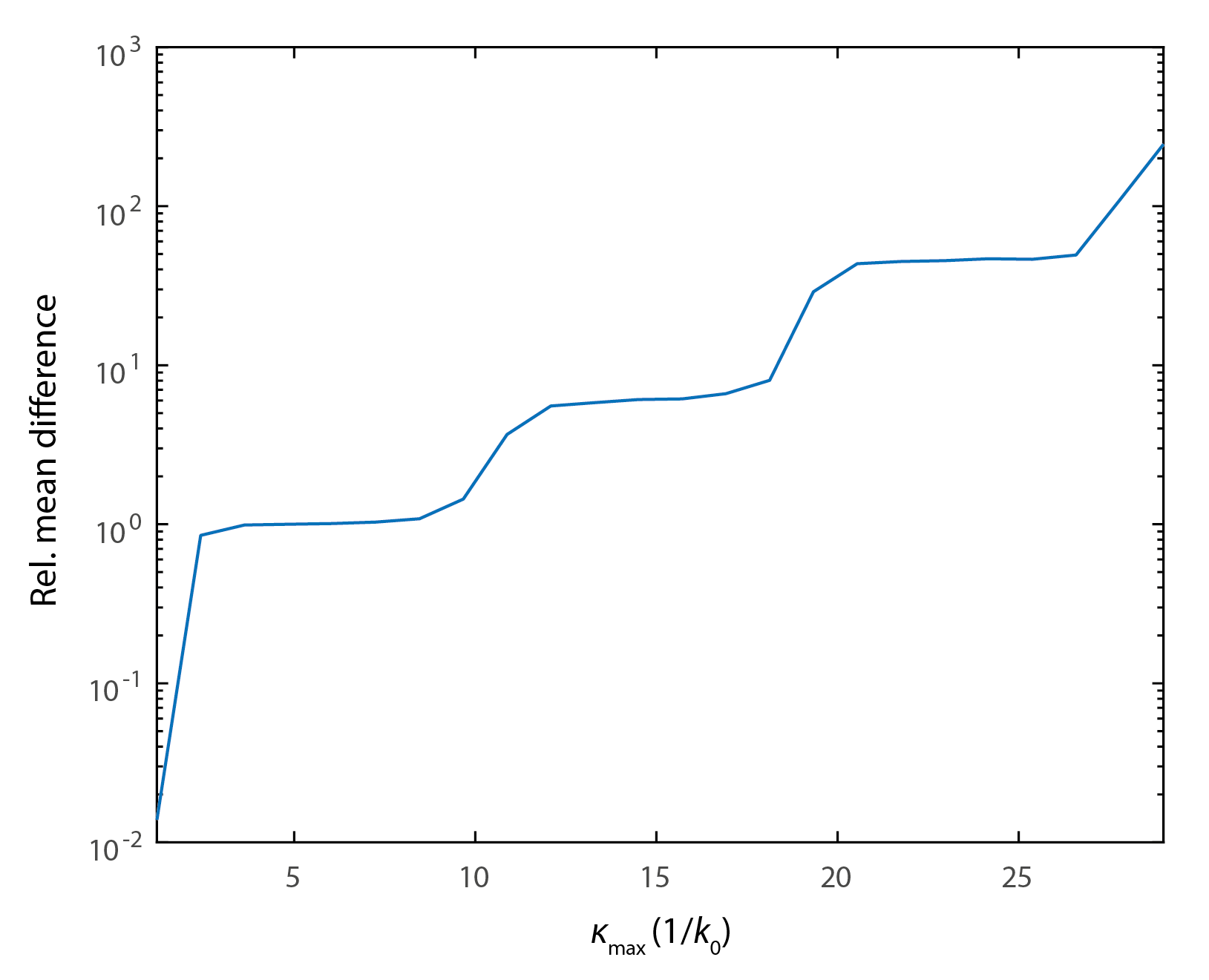


**Supplementary Figure S11: Mean difference dependence of filter value.** The mean is taken over all fields components and the y-axis is normalized to the difference when $\kappa_{\text{max}}=2k_{0}$, and is shown on a logarithmic scale. This figure was generated at a mean noise level of 0.2 of the maximum signal amplitude.

This observation is supported by Fig. S12, which shows the retrieved fields for $\kappa_{\text{max}}$ values directly before, on and beyond that first plateau. Evidently, $\kappa_{\text{max}}$ values before the plateau miss essential features in the retrieved fields. Likewise, values larger than the $\kappa_{\text{max}}$ corresponding to the first plateau ($\kappa_{\text{max}}\geq12k_{0}$) rapidly increase the noise in the retrieved fields, and clearly do not yield physically meaningful results. This empirical approach allows us to conclude that our choice of $\kappa_{\text{max}}=5k_{0}$ is correct (note that there is no qualitative difference between $\kappa_{\text{max}}=3k_{0}$ to $\kappa_{\text{max}}=9k_{0}$).


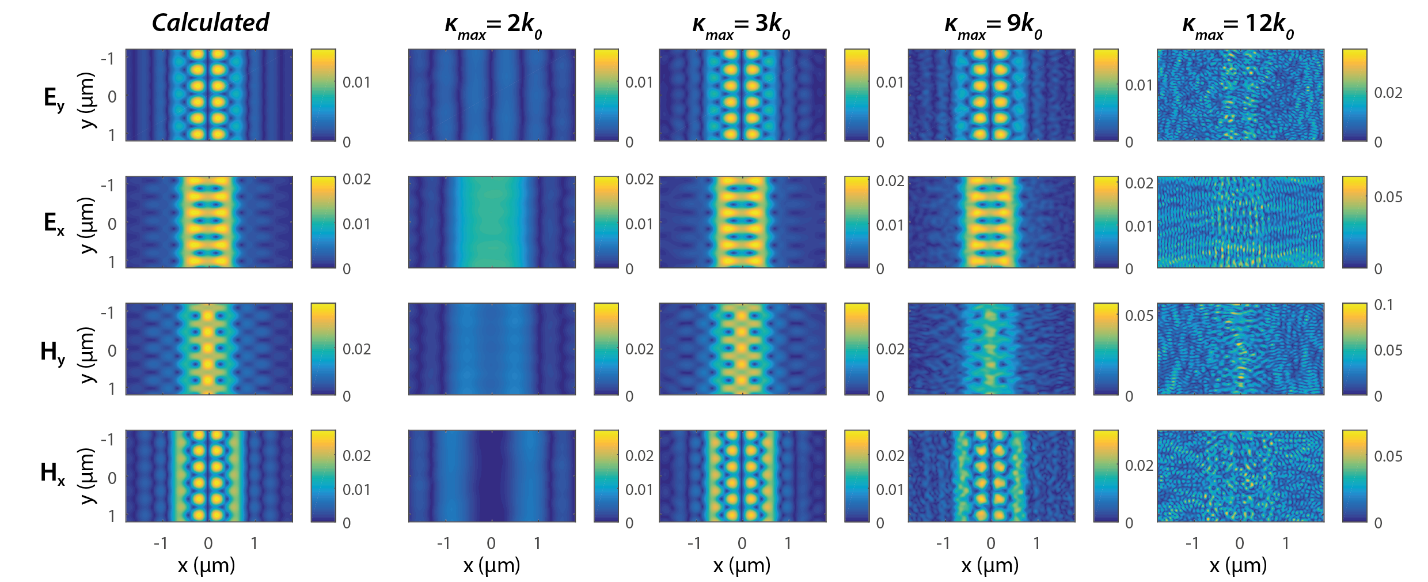


**Supplementary Figure S12: Effect of filtering on retrieved fields.** Panels show calculated retrieved electric and magnetic field maps for various filters, as indicated above each column. These panels were generated at a mean noise level of 0.2 of the maximum signal amplitude. Panel rows show the different electric and magnetic field components. The color bars next to each panel show the normalized field amplitude.

**
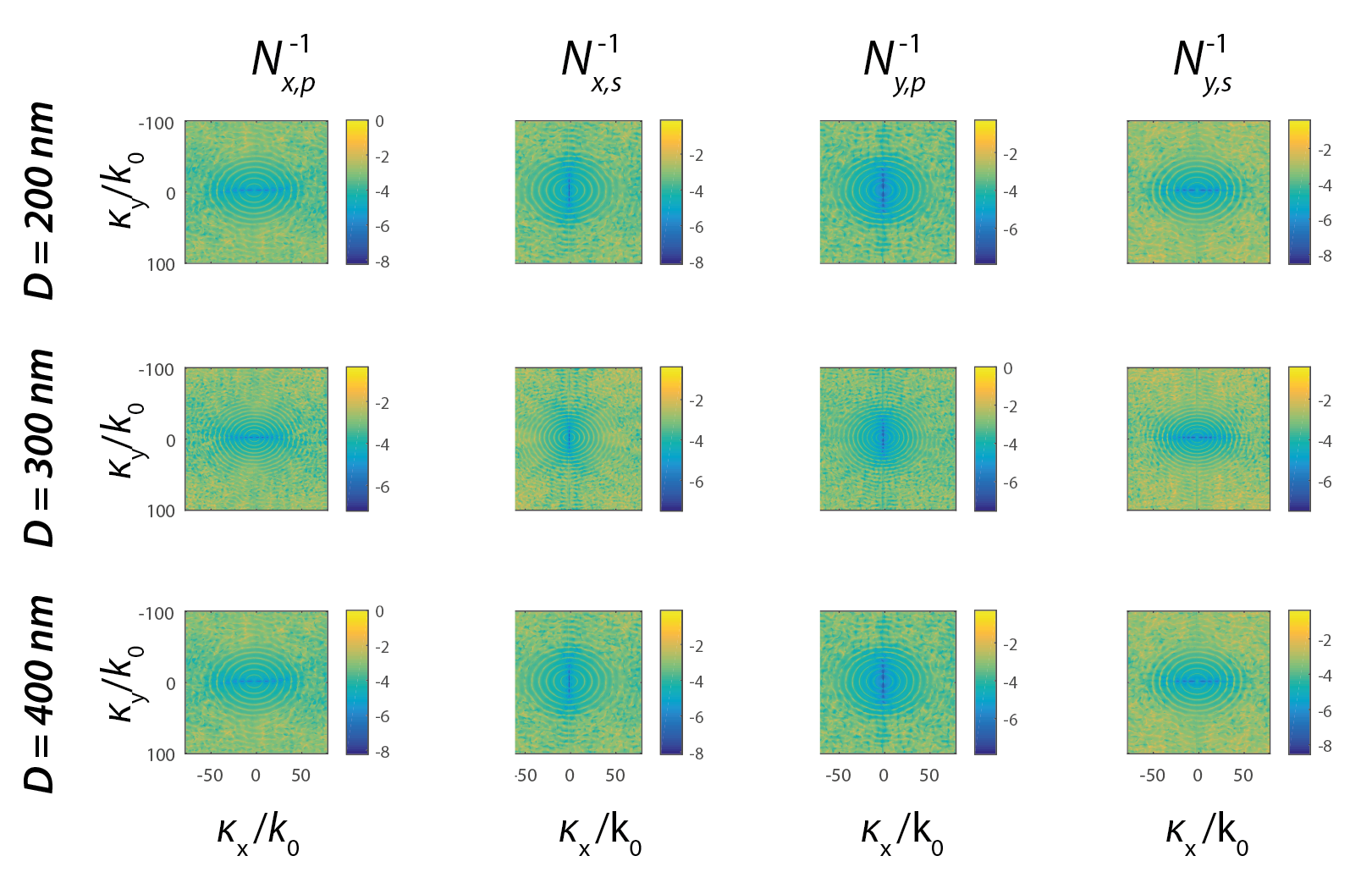
**

**Supplementary Figure S13: Effect of probe size on deconvolution matrix.** Each row of panels shows all components of log_10_(N^-1^) for the probe diameter (*D*) written next to that row. The component that each column represents is indicated above that column.


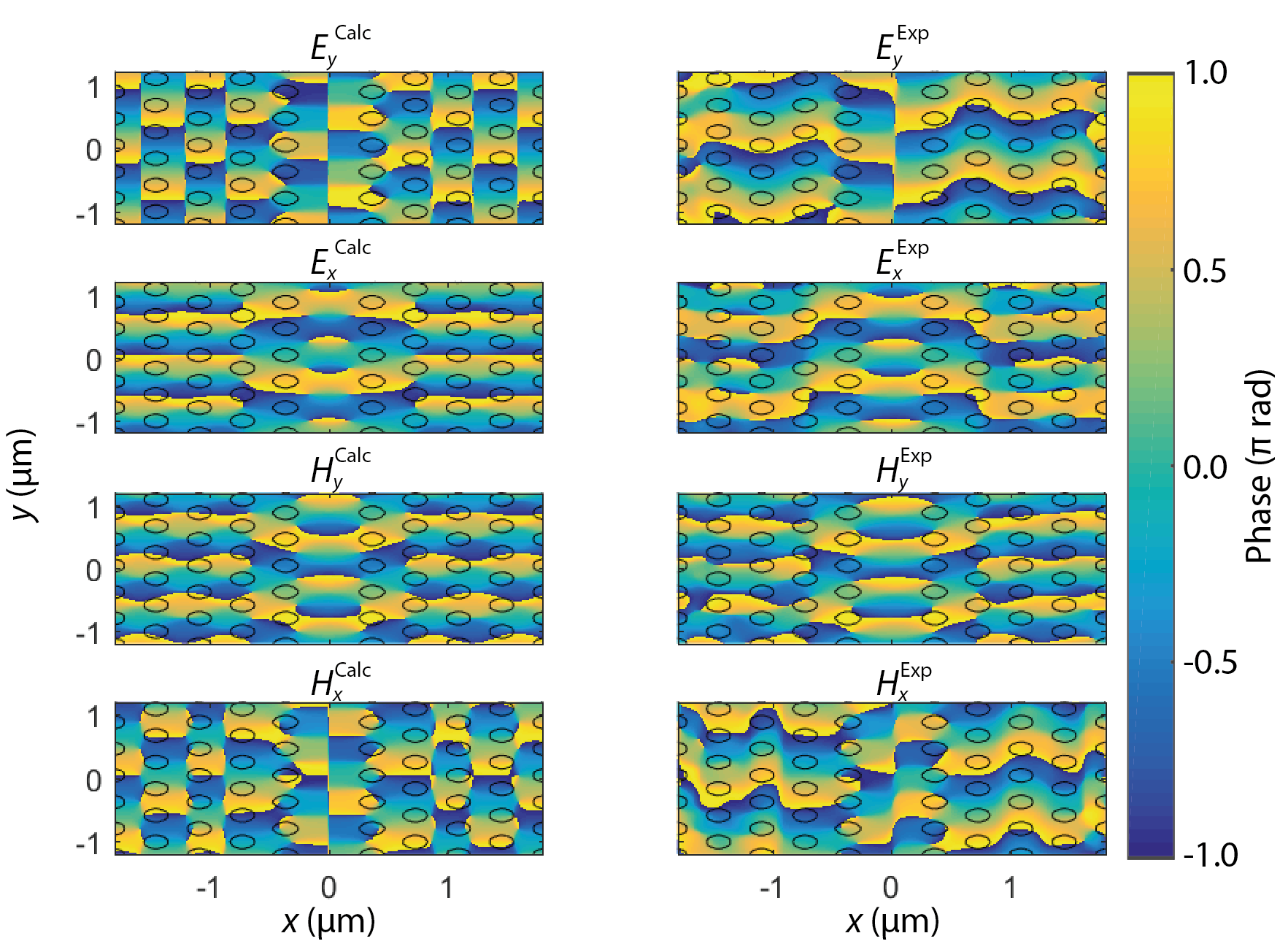


**Supplementary Figure S14: Retrieved PhCW phase maps.** The left (and right) column of panels show the calculated (and retrieved) phase maps of the electric and magnetic fields 280 nm above the PhCW. Each row of panels shows the component of the field indicated above the phase maps in that row.

1. le Feber, B.*, et al.* Simultaneous measurement of nanoscale electric and magnetic optical fields. Nat. Photon. **8**, 43-46 (2014)

2. Johnson, S.G. and Joannopoulos, J.D. Block-iterative frequency-domain methods for Maxwell’s equations in a planewave basis. Optics Express **8**, 173-190 (2001)

3. Verhagen, E.*, et al.* Nanowire Plasmon Excitation by Adiabatic Mode Transformation. Phys. Rev. Lett. **102**, 203904 (2009)

4. Johnson, P.B. and Christy, R.W. Optical Constants of the Noble Metals. Phys. Rev. B **6**, 4370-4379 (1972)

5. Kabakova, I.V.*, et al.* Imaging of electric and magnetic fields near plasmonic nanowires. Sci. Rep. **6**, 22665 (2016)

6. le Feber, B.*, et al.* Modal symmetries at the nanoscale: a route toward a complete vectorial near-field mapping. Opt. Lett. **39**, 2802-2805 (2014)
